# Exploring the role of genetic confounding in the association between maternal and offspring body mass index: evidence from three birth cohorts

**DOI:** 10.1093/ije/dyz095

**Published:** 2019-05-10

**Authors:** Tom A Bond, Ville Karhunen, Matthias Wielscher, Juha Auvinen, Minna Männikkö, Sirkka Keinänen-Kiukaanniemi, Marc J Gunter, Janine F Felix, Inga Prokopenko, Jian Yang, Peter M Visscher, David M Evans, Sylvain Sebert, Alex Lewin, Paul F O’Reilly, Debbie A Lawlor, Marjo-Riitta Jarvelin

**Affiliations:** 1 Department of Epidemiology and Biostatistics, School of Public Health, Imperial College London, London, UK; 2 Oulunkaari Health Center, Ii, Finland; 3 Medical Research Center, Oulu University Hospital and University of Oulu, Oulu, Finland; 4 Center for Life-Course Health Research, Faculty of Medicine, University of Oulu, Oulu, Finland; 5 Northern Finland Birth Cohort, Faculty of Medicine, University of Oulu, Oulu, Finland; 6 Healthcare and Social Services of Selänne, Pyhäjärvi, Finland; 7 Section of Nutrition and Metabolism, IARC, Lyon, France; 8 The Generation R Study Group, Erasmus MC, University Medical Center Rotterdam, Rotterdam, The Netherlands; 9 Department of Epidemiology, Erasmus MC, University Medical Center Rotterdam, Rotterdam, The Netherlands; 10 Department of Pediatrics, Erasmus MC, University Medical Center Rotterdam, Rotterdam, The Netherlands; 11 Section of Genomics of Common Disease, Department of Medicine, Imperial College London, London, UK; 12 Institute for Molecular Bioscience, University of Queensland, Brisbane, Australia; 13 Queensland Brain Institute, University of Queensland, Brisbane, Australia; 14 University of Queensland Diamantina Institute, Translational Research Institute, Brisbane, Australia; 15 MRC Integrative Epidemiology Unit at the University of Bristol, Bristol, UK; 16 Biocenter Oulu, University of Oulu, Oulu, Finland; 17 Department of Medical Statistics, London School of Hygiene and Tropical Medicine, London, UK; 18 MRC Social, Genetic and Developmental Psychiatry Centre, King’s College London, London, UK; 19 Population Health Science, Bristol Medical School, Bristol, UK; 20 Unit of Primary Care, Oulu University Hospital, Oulu, Finland; 21 Department of Life Sciences, College of Health and Life Sciences, Brunel University London, London, UK

**Keywords:** Maternal, offspring, BMI, genetic confounding, NFBCs, ALSPAC

## Abstract

**Background:**

Maternal pre-pregnancy body mass index (BMI) is positively associated with offspring birth weight (BW) and BMI in childhood and adulthood. Each of these associations could be due to causal intrauterine effects, or confounding (genetic or environmental), or some combination of these. Here we estimate the extent to which the association between maternal BMI and offspring body size is explained by offspring genotype, as a first step towards establishing the importance of genetic confounding.

**Methods:**

We examined the associations of maternal pre-pregnancy BMI with offspring BW and BMI at 1, 5, 10 and 15 years, in three European birth cohorts (*n* ≤11 498). Bivariate Genomic-relatedness-based Restricted Maximum Likelihood implemented in the GCTA software (GCTA-GREML) was used to estimate the extent to which phenotypic covariance was explained by offspring genotype as captured by common imputed single nucleotide polymorphisms (SNPs). We merged individual participant data from all cohorts, enabling calculation of pooled estimates.

**Results:**

Phenotypic covariance (equivalent here to Pearson’s correlation coefficient) between maternal BMI and offspring phenotype was 0.15 [95% confidence interval (CI): 0.13, 0.17] for offspring BW, increasing to 0.29 (95% CI: 0.26, 0.31) for offspring 15 year BMI. Covariance explained by offspring genotype was negligible for BW [–0.04 (95% CI: –0.09, 0.01)], but increased to 0.12 (95% CI: 0.04, 0.21) at 15 years, which is equivalent to 43% (95% CI: 15%, 72%) of the phenotypic covariance. Sensitivity analyses using weight, BMI and ponderal index as the offspring phenotype at all ages showed similar results.

**Conclusions:**

Offspring genotype explains a substantial fraction of the covariance between maternal BMI and offspring adolescent BMI. This is consistent with a potentially important role for genetic confounding as a driver of the maternal BMI–offspring BMI association.

**Key Messages**

- Maternal body mass index (BMI) is associated with offspring weight at birth and BMI in childhood and adulthood
- Each of these associations could be due to causal intrauterine effects, or confounding (genetic or environmental), or to some combination of these
- Our study suggests that a substantial part of the maternal BMI–offspring BMI association is explained by offspring genotype, but that in contrast the maternal BMI–offspring birth weight association is not explained by offspring genotype
- This is a first step towards establishing the importance of genetic confounding of the maternal BMI–offspring BMI association

## Introduction

It has been hypothesized that development in the uterus of an obese mother may programme a fetus for increased risk of obesity in subsequent postnatal life.^1–3^ Accordingly, intervening to prevent maternal obesity prior to pregnancy has been proposed as a means to reduce obesity risk in the offspring.^4–6^ Maternal body mass index (BMI) or obesity pre- or during pregnancy is associated with offspring adiposity measures at birth,^7^ in childhood^8–15^ and in adulthood,^16^^,^^17^ as well as offspring cardiometabolic risk factors and outcomes.^12^^,^^16^^,^^18–20^ However, these associations could be due to confounding, either by environmental factors or by maternal genotype inherited by the offspring. Furthermore, the contribution of causal intrauterine effects, genetic confounding and environmental confounding could be different for each of these associations.

Mendelian randomization (MR)^21^ evidence suggests that greater maternal BMI is likely to cause, via intrauterine mechanisms, greater offspring weight and ponderal index (PI) at birth.^22^ However, the balance of evidence from MR,^11^^,^^23^ within sibship analyses,^24^^,^^25^ and paternal negative exposure control studies^8–13^^,^^26^ suggests that maternal BMI is not causally related to offspring BMI in later life. It is therefore likely that confounding explains the association between maternal BMI and offspring child/adolescent adiposity but not offspring birth adiposity.

**Figure 1:**
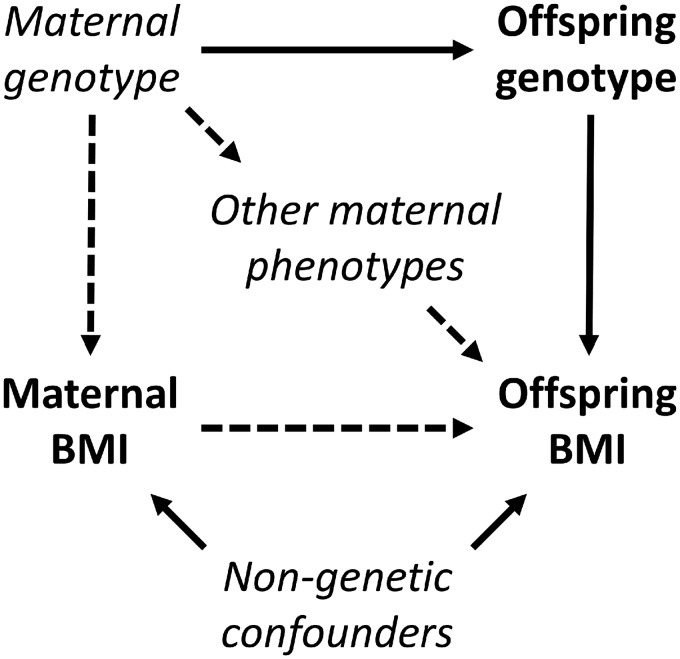
Directed acyclic graph (DAG) showing genetic confounding of the maternal BMI–offspring BMI association. The potentially causal association of interest is between maternal BMI and offspring BMI. The genetic confounding path (maternal BMI ← maternal genotype → offspring genotype → offspring BMI) results from direct effects of maternal genotype on maternal BMI and direct effects of offspring genotype on offspring BMI, as well as inheritance of maternal alleles by the offspring. We use the term genetic confounding to refer to only the aforementioned path; although another potential confounding path involves genotype (i.e. maternal BMI ← maternal genotype → other maternal phenotypes → offspring BMI), this latter path involves variables that are non-genetic from the offspring’s perspective. In the DAG, variables used in the present analysis are in bold lettering; other variables that we have not included in our analyses are italicized. Given that we include only offspring genotype, and not maternal genotype, in our analyses we are unable to distinguish genetic confounding from maternal genetic effects [i.e. indirect effects of maternal genotype on offspring BMI, mediated by the offspring’s prenatal or postnatal environment^40^ (dashed arrows)]; both could result in genetic covariance (Methods) between maternal BMI and offspring BMI.

In published studies adjustment for numerous potential confounders makes a negligible difference to the strength of the association between maternal (pre-)pregnancy adiposity and offspring adiposity in childhood or adulthood^9^^,^^11^^,^^12^^,^^24^^,^^26–35^ (Supplementary Note S1 and Supplementary Table S1, available as Supplementary data at *IJE* online). This could be because the confounders that were adjusted for were measured poorly, or because other unmeasured confounders explain the association; maternal genotype inherited by the offspring could be an important unmeasured confounder. General population data suggest that the narrow-sense heritability [the proportion of phenotypic variance due to additive genetic effects (denoted by *h*^2^)] of BMI is at least 30%,^36^^,^^37^ with higher estimates from family (∼45%) and twin (∼75%) studies.^38^^,^^39^ It is plausible therefore that the direct effects of alleles shared by the mother and offspring explain a substantial part of the maternal BMI–offspring BMI association; we refer to this as genetic confounding (Figure 1).

Here we aimed to estimate the extent to which the covariance between maternal BMI and offspring body size from birth to adolescence is explained by offspring genotype, as a first step towards establishing the importance of genetic confounding.

## Methods

### Study design

We analysed data from three prospective population-based birth cohorts: the Northern Finland Birth Cohort (NFBC) 1966,^41^ NFBC1986^42^ and Avon Longitudinal Study of Parents and Children (ALSPAC).^43^^,^^44^ Details of sample recruitment are given in Supplementary Note S2, available as Supplementary data at *IJE* online. Ethical approval for NFBC1966 and NFBC1986 was obtained from the University of Oulu Ethics Committee and the Ethical Committee of the Northern Ostrobothnia Hospital District, and for ALSPAC was obtained from the ALSPAC Ethics and Law Committee and the Local Research Ethics Committees.

### Exclusion criteria

We excluded stillbirths, multiple births and individuals with missing genotype data, and removed one member of any sibling pairs present at random. We then excluded participants with missing maternal BMI or offspring BMI/birth weight (BW) data. For our main analyses we used Genomic-relatedness-based Restricted Maximum Likelihood implemented in the GCTA software (GCTA-GREML), which requires that cryptic (unknown) relatedness be removed to avoid confounding due to familial environment and non-additive genetic effects.^45^ After merging data from the three cohorts we removed one individual from each cryptically related pair using a relatedness threshold of 0.05, resulting in inclusion of up to 11 498 participants (Supplementary Note S3 and Figure S4, available as Supplementary data at *IJE* online).

### Genotyping, quality control and imputation

Genotyping was carried out using genome-wide microarray chips followed by standard quality control (QC) procedures; details of genotyping and QC for each cohort are given in full in Supplementary Note S5, available as Supplementary data at *IJE* online. During QC, individuals with non-European ancestry were excluded. For all three cohorts, array genotypes were harmonized and imputed to the Haplotype Reference Consortium (HRC) imputation reference panel^46^ via the Michigan imputation server.^47^

### Maternal and offspring BW and BMI

For our primary analyses we examined the associations of maternal pre-pregnancy BMI with offspring weight at birth, and BMI at 1, 5, 10 and 15 years, in all studies (Supplementary Note S6, Table 1 and Supplementary Table S7, available as Supplementary data at *IJE* online). We also analysed BMI data at 31 and 46 years in NFBC1966. We calculated maternal pre-pregnancy BMI using pre-pregnancy weight reported by the mothers during early pregnancy and either self-reported or measured height (Supplementary Table S8, available as Supplementary data at *IJE* online). Offspring sex, BW, length and gestational age were obtained from the birth record or measured by research staff (Supplementary Table S8, available as Supplementary data at *IJE* online). In childhood and adulthood offspring weight and height were obtained from clinical examination, growth records or questionnaires (Supplementary Table S8, available as Supplementary data at *IJE* online). For all weight, height and BMI variables we set outlying values that we judged to be physiologically implausible to missing. We standardized maternal and offspring phenotypic variables to give mean zero and variance one in the pooled dataset, using the usual formula (Supplementary Note S9, available as Supplementary data at *IJE* online). With standardized variables, phenotypic covariance is equivalent to phenotypic correlation, enabling direct comparison of phenotypic covariance for offspring phenotypes that are measured in different units. Although BMI variables were positively skewed, sensitivity analyses indicated that results were similar when using a variety of normalizing transformations (Supplementary Note S10 and Figure S11, available as Supplementary data at *IJE* online), therefore we used untransformed variables for our primary analyses. Supplementary Note S12, available as Supplementary data at *IJE* online, gives details of other pregnancy variables that we used in sensitivity analyses.

**Table 1.** Phenotypic characteristics of the mothers and offspring. Sample sizes are the same as for the main analyses. Supplementary Note S39, available as Supplementary data at *IJE* online gives more detailed characteristics of the mothers and offspring.

Cohort	*n*		Phenotype			Age		Offspring sex	
			Mean	SD		Mean	SD	Male	Female
NFBC1966	2894	Maternal BMI (kg/m^2^)	23.0	3.3	Maternal age at offspring birth (years)	27.6	6.3		
NFBC1986	2094		22.2	3.3		28.0	5.3		
ALSPAC^a^	6510		22.9	3.8		29.4	4.6		
NFBC1966	2894	Birth weight (g)	3510	520	Gestational age at birth (weeks)	40.1	1.9	48.3%	51.7%
NFBC1986	2094		3610	490		40.0	1.5	49.3%	50.7%
ALSPAC^a^	6510		3450	520		39.5	1.7	51.2%	48.8%
NFBC1966	2736	1 year BMI (kg/m^2^)	17.8	1.6	Age at BMI measurement (years)	1.0	0.1	48.2%	51.8%
NFBC1986	1838		17.3	1.4		1.0	0.1	49.0%	51.0%
ALSPAC^a^	6159		17.5	1.5		0.9	0.2	51.2%	48.8%
NFBC1966	2145	5 year BMI (kg/m^2^)	15.5	1.4		5.1	0.8	49.4%	50.6%
NFBC1986	1840		15.8	1.5		5.0	0.4	49.2%	50.8%
ALSPAC^a^	5930		16.2	1.5		4.1	0.7	51.3%	48.7%
NFBC1966	2146	10 year BMI (kg/m^2^)	17.0	2.3		10.4	0.8	50.0%	50.0%
NFBC1986	1793		17.6	2.7		9.9	0.6	49.5%	50.5%
ALSPAC^a^	5494		17.7	2.8		9.9	0.5	50.2%	49.8%
NFBC1966	2866	15 year BMI (kg/m^2^)	19.7	2.6		14.7	0.5	48.0%	52.0%
NFBC1986	2107		21.3	3.7		16.0	0.4	48.6%	51.4%
ALSPAC^a^	4902		21.0	3.5		14.9	0.9	49.3%	50.7%
NFBC1966	3711	31 year BMI (kg/m^2^)	24.6	4.2		31.1	0.3	47.6%	52.4%
NFBC1966	3079	46 year BMI (kg/m^2^)	26.9	5.0		46.5	0.6	44.4%	55.6%

a ALSPAC offspring were born between 1991 and 1992.

SD, standard deviation.

### Estimation of genetic and residual covariance

We used bivariate GCTA-GREML to estimate the extent to which the phenotypic covariance between maternal BMI and offspring phenotype was explained by imputed offspring single nucleotide polymorphisms (SNPs). The simplest GCTA-GREML model is a univariate model^48^ that estimates the phenotypic variance explained by a set of genome-wide SNPs (termed the SNP heritability). Like other heritability estimation methods, GCTA-GREML exploits the fact that for heritable phenotypes, genetically similar individuals are likely to be phenotypically similar. Traditional heritability estimation methods use probability theory to infer expected genetic similarity between close relatives in pedigrees,^45^^,^^49^ and the phenotypic variance explained by all genetic variants is estimated. In contrast, in GCTA-GREML the genetic similarity between pairs of distantly related individuals is calculated directly from a set of SNPs, which enables utilization of non-pedigree samples. However, the phenotypic variance explained by only those genetic variants that are tagged by the set of SNPs is estimated. Accordingly, the two approaches estimate different quantities, and GCTA-GREML estimates are usually somewhat lower than pedigree-based heritability estimates.^36–39^ GCTA-GREML has been widely applied to diverse phenotypes.^37^^,^^50–53^

GCTA-GREML has been extended to a bivariate model that partitions the phenotypic covariance between two traits,^54^ and has again been widely applied to diverse phenotypes.^51^^,^^55–58^ Often these studies report the genetic correlation (*r*_G_) between two phenotypes, which quantifies the extent to which the additive genetic effects on phenotype one are shared with those on phenotype two (Supplementary Note S15, available as Supplementary data at *IJE* online). However, bivariate GCTA-GREML also enables estimation of the proportion of phenotypic covariance that is explained by the set of SNPs. This has previously been applied to two phenotypes measured in the same individual.^56^^,^^59^ In the present study we exploited this approach, but instead partitioned the phenotypic covariance between maternal BMI and offspring phenotype. In typical bivariate GCTA-GREML analyses, trait one, trait two and genotype are measured in the same individual, therefore the unit of analysis is the individual. In our analyses, genotype and trait one (offspring phenotype) were measured in the offspring and trait two (maternal BMI) was measured in the mother, therefore the unit of analysis was the mother–offspring dyad.

Assuming independence between additive genetic effects and other contributing factors, we can partition the phenotypic covariance as follows:

(Equation 1) $${\text{Cov}}_{\text{P}}={\text{ Cov}}_{\text{G}}+{\text{ Cov}}_{\text{E}}$$

where Cov_P_ is the covariance between maternal BMI and offspring phenotype (BW or BMI) estimated using the usual formula (Supplementary Note S9, available as Supplementary data at *IJE* online), Cov_G_ is the contribution to this covariance from additive genetic effects captured by the offspring’s imputed SNPs genome-wide, estimated using bivariate GCTA-GREML^54^ and Cov_E_ is the residual (unexplained) covariance, which is a combination of additive genetic effects not captured by SNPs, non-additive genetic effects and environmental effects (the latter would be referred to as common environmental effects in the quantitative genetics literature, because by definition common environmental effects are those that cause relatives to be more similar phenotypically). A detailed description of our statistical approach is given in Supplementary Note S9, available as Supplementary data at *IJE* online.

The ratio of Cov_G_ to Cov_P_ is our quantity of interest and has been termed the bivariate heritability^60^ or coheritability^61^ in the quantitative genetics literature. When both Cov_G_ and Cov_E_ have the same sign, Cov_G_:Cov_P_ is equivalent to the proportion of phenotypic covariance that is explained by additive genetic effects. If Cov_G_ and Cov_E_ are opposite in sign then Cov_G_:Cov_P_ may be negative or >1; in this case Cov_G_:Cov_P_ cannot be interpreted as a proportion, but still gives an indication of the extent to which phenotypic covariance is explained by genotype.

GCTA-GREML requires computation of a genetic relatedness matrix (GRM) containing a SNP-based estimate of relatedness for each pair of individuals in the sample. We used imputed autosomal SNPs with minor allele frequency (MAF) >0.01, imputation quality score (*r*^2^) >0.3 and lack of evidence for Hardy-Weinberg disequilibrium (*P*>1e-6); hard called (best-guess) genotypes (as output by the minimac3 software package^47^) were used to construct the GRM. Hard calls are integer values representing the most likely genotype, and are assigned by minimac3 based on the imputed haplotype probabilities. We fitted the GCTA-GREML model using a single GRM. Twenty ancestry informative principal components (PCs) calculated from the GRM were included as fixed effects in all models to adjust for population stratification; cohort, offspring sex and age at phenotype measurement (replaced with gestational age at birth for BW models) were also included as fixed effects.

We conducted sensitivity analyses (Supplementary Notes/Tables/Figures S10, S11 and S16–S33, available as Supplementary data at *IJE* online) to examine the impact of

1. alternative phenotype transformations including rank-based inverse-normal transformation, natural logarithm and UK-WHO *z*-scores
2. using different MAF and imputation *r*^2^ thresholds, as well as only directly genotyped (array) SNPs
3. varying the other covariates, as well as the number of PCs, that were fitted as fixed effects
4. varying the relatedness exclusion threshold
5. using alternative phenotypes including weight, BMI and PI [weight (kg)/height (m)^3^] at all ages.

We also tested for inflation of SNP heritability estimates due to cryptic relatedness or population stratification^62^^,^^63^ (Supplementary Note S34 and Supplementary Table S35, available as Supplementary data at *IJE* online). All analyses were performed using the GCTA software package^64^ version 1.91.1 with the ‘reml-no-constrain’ option; results were similar when we did not use this option.

### Estimation of confidence intervals and meta-analysis

The GCTA software supplies standard error (SE) estimates for Cov_G_, but not for Cov_G_:Cov_P_; we therefore used a leave-one-out jackknife procedure^65^^,^^66^ to estimate all SEs, and calculated 95% confidence intervals (CIs) as the point estimate ± 1.96 x SE (Supplementary Note S36, available as Supplementary data at *IJE* online). We confirmed via simulation that the jackknife approach is likely to give CIs with good coverage properties for a ratio of covariances (Supplementary Note S37, available as Supplementary data at *IJE* online). We merged individual participant data (IPD) from the three cohorts and fitted the GCTA-GREML model on this pooled dataset. In the meta-analysis literature this is referred to as one-stage IPD meta-analysis,^67^ and has also been referred to as mega-analysis, however for simplicity we use the term ‘pooled IPD estimates’ here. These pooled IPD estimates had greater statistical efficiency than a standard meta-analysis in which the GCTA-GREML model is fitted separately for each cohort, followed by estimation of the pooled effect using a fixed or random effects model. However, our pooled IPD estimates assumed that the three cohorts were from the same population. As a sensitivity analysis we therefore conducted a standard meta-analysis using a random effects model (DerSimonian and Laird^68^) which relaxed this assumption. Analyses were conducted in Stata version 13.1 (StataCorp, College Station, Houston, USA) and R version 3.5.0.^69^

## Results

### Sample characteristics

Table 1 shows the sample characteristics. Prevalence of maternal obesity (BMI≥30) was 3.7% (95% CI: 3.0%, 4.4%) in NFBC1966, 3.2% (95% CI: 2.4%, 3.9%) in NFBC1986 and 5.4% (95% CI: 4.9%, 6.0%) in ALSPAC. Maternal BMI was associated with several non-genetic potential confounders (Supplementary Table S39, available as Supplementary data at *IJE* online).

### Phenotypic and genetic covariance

**Figure 2:**
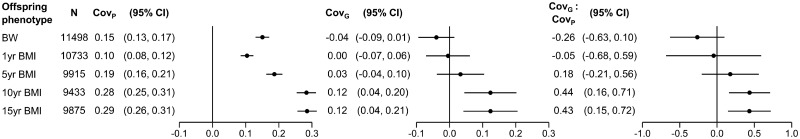
Estimates of phenotypic covariance (Cov_P_), genetic covariance (Cov_G_) and the ratio of Cov_G_ to Cov_P_, between maternal BMI and offspring phenotype, from the combined cohorts (pooled IPD estimates). All variables were standardized to give mean zero and variance one in the combined cohorts, therefore phenotypic covariances are equivalent to Pearson correlation coefficients. If Cov_G_ and Cov_E_ (the residual covariance) are opposite in sign then Cov_G_:Cov_P_ may be negative or >1; in this case Cov_G_:Cov_P_ cannot be interpreted as a proportion, but still gives an indication of the extent to which phenotypic covariance is explained by genotype. BW, birth weight, BMI, body mass index.

Table 2 shows correlations between maternal and offspring phenotypic variables. There were weak to moderate correlations between all phenotypes, with stronger correlations for temporally adjacent BMI phenotypes. Figure 2 shows pooled IPD estimates from the combined cohorts for the phenotypic covariance (Cov_P_), genetic covariance (Cov_G_) and the ratio of genetic to phenotypic covariance (Cov_G_:Cov_P_) between maternal BMI and offspring phenotype. Phenotypic covariance was 0.15 (95% CI: 0.13, 0.17) for offspring BW, decreasing to 0.10 (95% CI: 0.08, 0.12) for offspring 1 year BMI before increasing to 0.29 (95% CI: 0.26, 0.31) for offspring 15 year BMI. Covariance explained by offspring genotype was negligible for BW [–0.04 (95% CI: –0.09, 0.01)] but increased over childhood, reaching 0.12 (95% CI: 0.04, 0.20) at 10 years and 0.12 (95% CI: 0.04, 0.21) at 15 years, which is equivalent to 44% (95% CI: 16%, 71%) and 43% (95% CI: 15%, 72%) of the phenotypic covariance at 10 and 15 years respectively. This pattern continued into adulthood, with high Cov_G_:Cov_P_ estimated in NFBC1966 at 31 years [1.25 (95% CI: 0.35, 1.37)] and 46 years [0.78 (95% CI: –0.46, 1.87)], albeit with wide confidence intervals (Supplementary Table S40, available as Supplementary data at *IJE* online).

**Table 2.** Correlation matrices for maternal and offspring phenotypic variables. Values are Pearson correlation coefficients

Cohort	Phenotype	Birth weight	1 year BMI	5 year BMI	10 year BMI	15 year BMI	31 year BMI	46 year BMI
NFBC1966	Maternal BMI	0.22	0.13	0.16	0.22	0.22	0.18	0.16
	Birth weight		0.22	0.20	0.15	0.11	0.06	0.06
	1 year BMI			0.49	0.32	0.27	0.17	0.12
	5 year BMI				0.66	0.53	0.35	0.26
	10 year BMI					0.77	0.50	0.40
	15 year BMI						0.58	0.49
	31 year BMI							0.80
NFBC1986	Maternal BMI	0.19	0.09	0.19	0.25	0.27		
	Birth weight		0.18	0.18	0.13	0.08		
	1 year BMI			0.53	0.34	0.22		
	5 year BMI				0.75	0.61		
	10 year BMI					0.77		
ALSPAC	Maternal BMI	0.13	0.09	0.19	0.32	0.35		
	Birth weight		0.20	0.18	0.13	0.10		
	1 year BMI			0.44	0.25	0.20		
	5 year BMI				0.50	0.39		
	10 year BMI					0.79		

### Sensitivity analyses

Standard meta-analysis using a random effects model gave similar estimates to the pooled IPD estimates, although with wider confidence intervals (Supplementary Notes/Tables/Figures S41–S47, available as Supplementary data at *IJE* online), and estimates changed little as we varied covariates, phenotypes (weight, BMI or PI) or normalizing transformations (Supplementary Notes/Figures S10, S11, S20, S30–S33, available as Supplementary data at *IJE* online). Results from analyses in which we varied the relatedness exclusion threshold or the set of SNPs used to calculate the GRM suggested that our primary analyses are unlikely to be substantively biased, and estimates for Cov_G_:Cov_P_ and SNP heritability were not attenuated as we varied the number of PCs fitted as fixed effects between zero and one thousand (Supplementary Notes/Tables/Figures S16–S29, available as Supplementary data at *IJE* online). Finally, we fitted the univariate GCTA-GREML model with disjoint halves of the genome and found little evidence of inflation of SNP heritability estimates due to cryptic relatedness or population stratification (Supplementary Note S34 and Supplementary Table S35, available as Supplementary data at *IJE* online).

## Discussion

### Main findings

We estimate that offspring genotype, as captured by common imputed SNPs, explains 43% of the covariance between maternal pre-pregnancy BMI and offspring 15 year BMI. In contrast, offspring genotype does not explain the covariance between maternal BMI and offspring BW, although we could not reject the possibility of a small genetic covariance here due to the imprecision of the estimate. The observed pattern of genetic covariance is consistent with the hypothesis that maternal alleles inherited by the offspring potentially have an important confounding effect on the association between maternal BMI and offspring child and adolescent BMI. However, further work using methods that account for maternal genotype^70^ will be required before this conclusion can be drawn.

### Interpretation

To our knowledge we are the first to use bivariate GCTA-GREML to partition the covariance between the same phenotype measured in the mother and offspring, although the method has previously been used to investigate genetic covariance between offspring BW and cardiometabolic traits^56^ and family socio-economic position and offspring educational attainment.^59^ Genetic covariance was close to zero for maternal BMI and offspring BW, suggesting that genetic confounding (Figure 1) does not explain this association. This is consistent with MR evidence,^22^ paternal negative exposure control studies,^9^^,^^13^^,^^71^^,^^72^ and evidence of minimal shared genetic aetiology between BW and adult BMI.^56^ In contrast, offspring genotype explained almost half of the covariance between maternal BMI and offspring BMI in late childhood and adolescence, which is consistent with an important role for genetic confounding for this latter association. However, our present data are insufficient to firmly draw this conclusion: because of the correlation between offspring genotype and maternal genotype, our estimate of genetic covariance could include a contribution from any effects of maternal genotype on offspring BMI via the offspring’s prenatal or postnatal environment, including any causal intrauterine effect. Data from a recent study suggest that parental BMI-increasing genotype does not have a large indirect effect on offspring BMI via the offspring’s environment,^73^ which in combination with our data would suggest an important role for genetic confounding, consistent with MR,^11^^,^^23^ within sibship analyses,^24^^,^^25^ and paternal negative exposure control studies.^8–13^^,^^26^ In future work it will be important use the maternal GCTA-GREML model^70^ to test for maternal genetic effects on childhood BMI, which if absent would provide more evidence for the presence of genetic confounding when considered in combination with our present results. It should also be noted that our estimate of genetic covariance only takes into account genetic variation captured by common imputed SNPs, and therefore represents a lower bound on the true genetic covariance.

Simulation studies suggest that the GCTA-GREML model is robust to violation of several of its assumptions.^74^ However, GCTA-GREML estimates can be biased if causal genetic variants have dissimilar MAF or linkage disequilibrium (LD) properties to the SNPs used to calculate the GRM.^36^^,^^62^^,^^74^^,^^75^ A recent simulation study by Evans *et al.*^37^ concluded that MAF stratified (MS) or LD and MAF stratified (LDMS) GCTA-GREML models are most robust to these potential biases; unfortunately we had insufficient sample size to implement GCTA-GREML-MS or GCTA-GREML-LDMS. However, we are reassured by the empirical results presented by Evans *et al.*: in the UK Biobank single-component-GCTA-GREML (GCTA-GREML-SC) using imputed SNPs with MAF >0.01 gave a similar SNP heritability estimate for BMI to the gold standard GCTA-GREML-LDMS-I model.^37^ Given that we used SNPs with MAF >0.01 for our primary GCTA-GREML-SC analyses, it seems unlikely that our estimates for the ratio of genetic to phenotypic covariance are substantively affected by MAF or LD related biases.

### Strengths and limitations

Our study has several important strengths. We analysed rich prospective data from three birth cohorts, collected from early pregnancy to adolescence (and until middle age in one study). Our use of bivariate GCTA-GREML enabled inference on the combined effects of hundreds or thousands of genetic variants that individually would not be observable. Furthermore, we meta-analysed data from three cohorts, giving sufficient sample size to obtain statistically robust evidence for genetic covariance. However, replication in other birth cohorts would be desirable, particularly as the mothers in our cohorts were lean compared with many present-day populations in high-income countries.^5^ Our primary pooled IPD estimates were not meaningfully changed when we instead used standard meta-analysis with a random effects model, relaxing the assumption of effect homogeneity (Supplementary Notes/Tables/Figures S41–S47, available as Supplementary data at *IJE* online). We conducted extensive sensitivity analyses to explore the likelihood of bias due to confounding by familial environment^45^ or population stratification^76^^,^^77^ (Supplementary Notes/Tables/Figures S20–S29, S34 and S35, available as Supplementary data at *IJE* online). Given reassuring results from analyses in which we (i) varied the relatedness exclusion threshold, (ii) fitted a large number of principal components as fixed effects, and (iii) used disjoint halves of the genome to test for inflation due to population structure, we feel that neither coarse nor fine population structure are likely to pose a serious threat to the validity of our findings.

Several limitations apply to this work. First, assortative mating has been observed for BMI,^78^ and the implications for heritability estimation using GCTA-GREML are currently unclear. Second, selection bias may occur even in studies such as ours that estimate genetic effects.^79^ We note that associations between maternal BMI and offspring BW were similar in the samples used for our main analyses and the larger sample of live born babies at baseline (Supplementary Note S48 and Supplementary Table S49, available as Supplementary data at *IJE* online), suggesting that this phenotypic association is unlikely to be meaningfully affected by selection bias. Although we are unable to rule out an effect of selection bias on our genetic covariance estimates, it seems unlikely that such an effect would be of sufficient magnitude to wholly account for our results. Finally, weight at birth and BMI from childhood to adulthood are imperfect proxy measures for adiposity. However, there is evidence that the correlation with directly measured adiposity is strong for child and adult BMI^80^^,^^81^ and moderate for neonatal weight.^82^

### Conclusion

In conclusion, our data are consistent with, although do not confirm, the hypothesis that genetic confounding explains a substantial part of the association between maternal pre-pregnancy BMI and offspring adolescent BMI. It will be important to confirm whether this is the case, because if there is substantial genetic confounding then intervention to reduce maternal pre-pregnancy BMI with the aim of reducing offspring obesity risk will have a smaller effect than if such confounding did not exist.

## Funding

NFBC1966 and 1986 have received financial support from the Academy of Finland [EGEA, grant number: 285547]; University Hospital Oulu, Biocenter, University of Oulu, Finland [grant number: 75617; 2016-20]; NIHM [grant number: MH063706]; Juselius Foundation; NHLBI [grant number: 5R01HL087679-02] through the STAMPEED program [grant number: 1RL1MH083268-01]; the European Commission [EURO-BLCS, Framework 5 award QLG1-CT-2000-01643], the Medical Research Council, UK [grant numbers: MR/M013138/1, MRC/BBSRC, MR/S03658X/1 (JPI HDHL)]; the EU H2020 DynaHEALTH action [grant number: 633595]; the EU H2020-HCO-2004 iHEALTH Action [grant number: 643774]; the EU H2020-PHC-2014 ALEC Action [grant number: 633212]; the EU H2020-SC1-2016-2017 LifeCycle Action [grant number: 733206]; the EU H2020-MSCA-ITN-2016 CAPICE Action [grant number: 721567]. The DNA extractions, sample quality controls, biobank upkeep and aliquoting were performed in the National Public Health Institute, Biomedicum Helsinki, Finland and supported financially by the Academy of Finland and Biocentrum Helsinki. The UK Medical Research Council and Wellcome [grant number: 102215/2/13/2] and the University of Bristol provide core support for ALSPAC. Genotyping of the ALSPAC maternal samples was funded by the Wellcome Trust [grant number: WT088806] and the offspring samples were genotyped by Sample Logistics and Genotyping Facilities at the Wellcome Trust Sanger Institute and LabCorp (Laboratory Corporation of America) using support from 23andMe. This study was also supported by the US National Institute of Health [grant number: R01 DK10324] and the European Research Council under the European Union’s Seventh Framework Programme (FP7/2007–2013) / ERC grant agreement [grant number: 669545]. A comprehensive list of grants funding is available on the ALSPAC website (http://www.bristol.ac.uk/alspac/external/documents/grant-acknowledgements.pdf). T.A.B. is supported by the Medical Research Council (UK) [grant number: MR/K501281/1]. D.M.E. and D.A.L. work in / are affiliated with a unit that is supported by the UK Medical Research Council [grant number: MC_UU_00011/6] and D.A.L. is a NIHR Senior Investigator [grant number: NF-SI-0611–10196]. I.P. is funded by the World Cancer Research Fund (WCRF UK) and World Cancer Research Fund International [grant number: 2017/1641] and the Wellcome Trust [grant number: WT205915].

This publication is the work of the authors and T.A.B., M.-R.J. and D.A.L. will serve as guarantors for the contents of this paper.

## Supplementary Material

dyz095_Supplementary_Data
