# Supplementary material for: Exploring the role of genetic confounding in the association between maternal and offspring body mass index: evidence from three birth cohorts
Source: Int J Epidemiol. 2019 May 10;49(1):233–43. doi: 10.1093/ije/dyz095 (PMC7245052; doi:10.1093/ije/dyz095)
Supplement: dyz095_Supplementary_Data [file dyz095_supplementary_data.pdf]

Exploring the role of genetic confounding in the association between maternal and offspring body mass index: evidence from three birth cohorts

Supplementary Information

Tom A. Bond[1]*, Ville Karhunen[1], Matthias Wielscher[1], Juha Auvinen[2, 3, 4], Minna Männikkö[5], Sirkka Keinänen-Kiukaanniemi[3, 4, 6], Marc J. Gunter[7], Janine F. Felix[8, 9, 10], Inga Prokopenko[11], Jian Yang[12, 13], Peter M. Visscher[12, 13], David M. Evans[14, 15], Sylvain Sebert[5, 16], Alex Lewin[1, 17], Paul F. O'Reilly[1, 18], Debbie. A. Lawlor[† 15, 19], Marjo-Riitta Jarvelin[† 1, 5, 16, 20, 21].

* Corresponding author.

[†] These authors contributed equally to the work.

Affiliations: *1: Department of Epidemiology and Biostatistics, School of Public Health, Imperial College London, London, UK. 2. Oulunkaari Health Center, Ii, Finland. 3. Medical Research Center, Oulu University Hospital and University of Oulu, Oulu, Finland. 4: Center for Life-Course Health Research, Faculty of Medicine, University of Oulu, Oulu, Finland. 5: Northern Finland Birth Cohort, Faculty of Medicine, University of Oulu, Oulu, Finland. 6: Healthcare and Social Services of Selänne, Pyhäjärvi, Finland 7: Section of Nutrition and Metabolism, IARC, Lyon, France. 8: The Generation R Study Group, Erasmus MC, University Medical Center Rotterdam, Rotterdam, The Netherlands. 9: Department of Epidemiology, Erasmus MC, University Medical Center Rotterdam, Rotterdam, The Netherlands. 10: Department of Pediatrics, Erasmus MC, University Medical Center Rotterdam, Rotterdam, The Netherlands. 11: Section of Genomics of Common Disease, Department of Medicine, Imperial College London, London, UK. 12: Institute for Molecular Bioscience, University of Queensland, Brisbane, Australia. 13: Queensland Brain Institute, University of Queensland, Brisbane, Australia. 14: University of Queensland Diamantina Institute, Translational Research Institute, Brisbane, Australia. 15: MRC Integrative Epidemiology Unit at the University of Bristol, Bristol, UK. 16: Biocenter Oulu, University of Oulu,*

Oulu, Finland. 17: Department of Medical Statistics, London School of Hygiene and Tropical Medicine, London, UK. 18: MRC Social, Genetic and Developmental Psychiatry Centre, King's College London, London, UK. 19: Population Health Science, Bristol Medical School, Bristol, UK. 20: Unit of Primary Care, Oulu University Hospital, Oulu, Finland. 21: Department of Life Sciences, College of Health and Life Sciences, Brunel University London, London, UK.

Table of contents

Exploring the role of genetic confounding in the association between maternal and offspring body mass index: evidence from three birth cohorts 1

Note S1: Published phenotypic associations between maternal pre-pregnancy adiposity and offspring adiposity from childhood to adulthood 4

Note S2: Cohort sample descriptions 10

Note S3: Sample derivation flow charts 10

Figure S4: Sample derivation flow charts 11

Figure S4 (continued): Sample derivation flow charts 12

Figure S4 (continued): Sample derivation flow charts 13

Figure S4 (continued): Sample derivation flow charts 14

Note S5: Genotyping and quality control 14

NFBC1966 14

NFBC1986 14

ALSPAC 15

Note S6: Selection of offspring anthropometric measurements within age windows 15

Table S8: Source of anthropometric measurements 16

Note S9: Equations for phenotypic variable standardisation and phenotypic covariance, and statistical details of the GCTA-GREML model 17

Phenotypic variable standardisation 17

Phenotypic covariance 17

GCTA-GREML 17

Note S10: Sensitivity analysis 1—Phenotypic variable transformations 19

Note S12: Source of other variables 20

NFBC1966 20

NFBC1986 21

ALSPAC 21
Note S13: Univariate GCTA-GREML estimates of SNP heritability 22
Note S15: Relationship of bivariate heritability to genetic correlation 22
Note S16: Sensitivity analysis 2—SNPs used to calculate the genetic relatedness matrix.....23
Note S20: Sensitivity analysis 3—Number of principal components and other covariates 26
Note S25: Sensitivity analysis 4—Relatedness exclusion threshold 30
Note S30: Sensitivity analysis 5—Alternative phenotypes 33
Note S34: Testing for inflation of heritability estimates due to population structure 35
Note S36: Leave-one-out jackknife procedure 35
Note S37: Validation of jackknife procedure via simulation 36
Table S39: Associations between maternal BMI, potential confounders and offspring phenotypes....................................38
Table S40: Phenotypic and genetic covariance at all ages in the combined cohorts.............40
Note S41: Standard meta-analysis results....................................40
Note S48: Examining the effect of missing data....................................45
Table S50: Number of individuals removed at varying relatedness exclusion thresholds.......47
Abbreviations....................................48
References....................................48

Note S1: Published phenotypic associations between maternal pre-pregnancy adiposity and offspring adiposity from childhood to adulthood

We conducted a literature search with the aim of identifying studies that examined the association between maternal pre-pregnancy adiposity and offspring adiposity in childhood or adulthood, and that presented results both unadjusted and adjusted for potential confounders. We used the following Medline search terms:

1. maternal
2. **AND** pregnancy
3. **AND** BMI **OR** body mass index
4. **AND** offspring **OR** child

We did not limit results by year of publication. The search returned 2693 results, which we assessed against the following inclusion and exclusion criteria:

Inclusion criteria:

- Studies which presented a regression coefficient for the association between a continuous measure of maternal adiposity (pre-pregnancy or during pregnancy) and a continuous measure of offspring adiposity (at age > 1 year), both adjusted and unadjusted for potential confounders

Exclusion criteria

- Studies which adjusted for offspring body size variables (e.g. birth weight, childhood body mass index (BMI)), which are potential mediators
- Studies which only presented results from analysis treating exposure and/or outcome as categorical variables
- Studies for which the outcome was change in adiposity (as opposed to absolute adiposity level)

After screening titles and abstracts against the criteria above, 64 studies remained. After assessing the full text of articles, 14 studies remained, and are presented in **Table S1** below.

Based on triangulated evidence from several study designs it is likely that confounding is an important driver of the maternal adiposity-offspring child/adolescent adiposity association (**Main Text**). The studies that we have identified here carried out adjustment for a variety of potential confounders, and in almost all studies this adjustment had a negligible impact on the magnitude of the association. There are two possible explanations for this finding: first, that confounders were measured poorly, and second, that other unmeasured confounders (for example genotype) have an important effect.

Table S1: Published phenotypic associations between maternal adiposity and offspring adiposity, unadjusted and adjusted for potential confounders. **a**: Regression coefficient from linear regression of offspring outcome on maternal exposure, with covariates as described in the table. **b**: Ratio of the regression coefficient from the fully adjusted model to that from the minimally adjusted model

**Cohort**	**Year of birth**	**N**	**Maternal exposure**	**Offspring outcome**	**Covariates**	**β^a (95% CI)**	**$\beta_{FULL} : \beta_{MINIMAL}$[b]**
Uppsala Birth Cohort (1)	1983-1985	1103	Pre-pregnancy BMI (kg/m^2), from birth registry	18 year BMI (kg/m^2), clinically measured	Offspring age	0.38 (0.29, 0.47)	1.03
					As above, plus maternal age, parity, education and smoking	0.39 (0.30, 0.47)	
Fukoroi, Japan (2)	1998-1999	480	Early pregnancy BMI (kg/m^2), abstracted from health records	13 year BMI (*z*-score), measured by teachers	None	Males: 0.14 ($P = 0.029$), Females: 0.47 ($P < 0.001$)	Males: 1.07, Females: 1.04
					Gestational age, maternal age at delivery, smoking during pregnancy	Males: 0.15 ($P = 0.015$), Females: 0.49 ($P < 0.001$)	
Born in Bradford (3)	2007-2010	6060	Early pregnancy BMI (12 weeks gestation) (kg/m^2), abstracted from antenatal records	4-5 year BMI (kg/m^2), clinically measured	Child age and sex	White British: 0.06 (0.05, 0.07), Pakistani: 0.09 (0.08, 0.10)	White British: 1.17, Pakistani: 1.11
					As above, plus maternal age, parity, education, smoking, housing tenure and whether anyone in the household was in receipt of means tested benefits	White British: 0.07 (0.05, 0.08), Pakistani: 0.10 (0.09, 0.11)	
Mysore Parthenon Study (4)	1997-1998	504	Late pregnancy sum of skinfolds (30 weeks gestation) (*z*-score), clinically measured	9.5 year BMI (kg/m^2), clinically measured	None	0.52 (0.36, 0.68)	0.94
					Offspring age, sex, breast-feeding duration, maternal age, parity and socioeconomic position	0.49 (0.32, 0.65)	

Table S1 (continued): Published phenotypic associations between maternal adiposity and offspring adiposity, unadjusted and adjusted for potential confounders. **a**: Regression coefficient from linear regression of offspring outcome on maternal exposure, with covariates as described in the table. **b**: Ratio of the regression coefficient from the fully adjusted model to that from the minimally adjusted model

Cohort	Year of birth	N	Maternal exposure	Offspring outcome	Covariates	β^a (95% CI)	$\beta_{FULL} : \beta_{MINIMAL}$[b]
Columbia Center for Children's Environmental Health Mothers and Newborns Study (5)	1998-2006	323	Pre-pregnancy BMI (kg/m^2), calculated using self reported weight and self reported or clinically measured height	7 year BMI (CDC *z*-score), clinically measured	Gestational age, gestational weight gain	0.26 (0.16, 0.35)	1.12
					As above, plus parity, offspring age, sex, maternal ethnicity, maternal education, receipt of public assistance, prenatal demoralisation score and ability to afford food in pregnancy	0.29 (0.19, 0.39)	
Mater-University of Queensland Study of Pregnancy (MUSP) (6)	1981-1983	2229	Pre-pregnancy BMI (kg/m^2), calculated using self reported weight and clinically measured height	21 year BMI (kg/m^2), clinically measured	None	0.35 (0.31, 0.40)	1.09
					Gestational weight gain, maternal age at birth, maternal smoking during pregnancy, maternal and paternal education, family income	0.38 (0.32, 0.43)	
Collaborative Perinatal Project (7)	1959-1965	33,893	Pre-pregnancy BMI (kg/m^2), calculated using self reported weight and clinically measured height	7 year BMI (kg/m^2), clinically measured	None	0.05 (0.05, 0.06)	1.20
					Maternal age, education, race, smoking during pregnancy, parity, offspring sex, age, age2, preterm birth and hospital site	0.06 (0.06, 0.07)	

Table S1 (continued): Published phenotypic associations between maternal adiposity and offspring adiposity, unadjusted and adjusted for potential confounders. **a**: Regression coefficient from linear regression of offspring outcome on maternal exposure, with covariates as described in the table. **b**: Ratio of the regression coefficient from the fully adjusted model to that from the minimally adjusted model

Cohort	Year of birth	N	Maternal exposure	Offspring outcome	Covariates	β^a (95% CI)	$\beta_{FULL} : \beta_{MINIMAL}{}^b$
2004 Pelotas Birth Cohort (8)	2004	3123 (unadjusted)<br><br>3064 (fully adjusted)	Pre-pregnancy BMI (kg/m^2), calculated using weight from the prenatal record or self reported weight, and clinically measured height	6 year fat mass index (kg/m^2), measured using air displacement plethysmography	None	0.11 (0.10, 0.13)	0.64
					Family income, skin colour, maternal age, maternal education, parity, child's sex and pre-pregnancy BMI x maternal education interaction term	0.07 (0.02, 0.11)	
Amsterdam Born Children and their Development Cohort (ABCD) (9)	2003-2004	1727	Pre-pregnancy BMI (kg/m^2), calculated using self reported weight and height	5-6 year body fat percentage (bioelectrical impedance)	Offspring age and sex	0.32 (0.24, 0.40)	0.66
					As above, plus maternal age, parity, ethnicity, maternal height, maternal years of education, maternal alcohol intake, maternal smoking, maternal hypertension, duration of exclusive breastfeeding, offspring screen time and offspring saturated fat intake	0.21 (0.13, 0.29)	

Table S1 (continued): Published phenotypic associations between maternal adiposity and offspring adiposity, unadjusted and adjusted for potential confounders. **a**: Regression coefficient from linear regression of offspring outcome on maternal exposure, with covariates as described in the table. **b**: Ratio of the regression coefficient from the fully adjusted model to that from the minimally adjusted model

Cohort	Year of birth	N	Maternal exposure	Offspring outcome	Covariates	β[a] (95% CI)	$\beta_{FULL} : \beta_{MINIMAL}$[b]
The Generation R Study (10)	2002-2006	4871	Pre-pregnancy BMI (*z*-score), calculated using self reported weight and clinically measured height	6 year BMI (*z*-score), clinically measured	Sex, offspring age, paternal BMI	0.21 (0.18, 0.24)	1.00
					As above, plus maternal age, education, ethnicity, parity, smoking and alcohol consumption during pregnancy, folic acid supplementation, calorie intake, caesarean delivery, breast feeding, solid food timing, offspring television watching duration	0.21 (0.18, 0.24)	
Norwegian Mother and Child Cohort Study (MoBa) (11)	2001-2007	29,216	Pre-pregnancy BMI (*z*-score), calculated using self reported weight and height	3 year BMI (*z*-score), calculated using maternal report of offspring weight and height	Paternal BMI	0.12 (0.10, 0.14)	1.00
					As above, plus parental education, parental prenatal smoking, maternal coffee consumption, number of siblings, day care, breastfeeding, outdoor activities, watching television/video, diet, parental postnatal smoking	0.12 (0.10, 0.14)	
Swedish record linkage (12)	1973-1988	146,894	Early pregnancy BMI (around 10 weeks gestation) (kg/m^2), abstracted from birth records	18 year BMI (kg/m^2), clinically measured during conscription examination	Year of birth	0.31 (0.30 to 0.31)	0.97
					As above, plus maternal age at birth, parity and education	0.30 (0.29 to 0.31)	

Table S1 (continued): Published phenotypic associations between maternal adiposity and offspring adiposity, unadjusted and adjusted for potential confounders. **a**: Regression coefficient from linear regression of offspring outcome on maternal exposure, with covariates as described in the table. **b**: Ratio of the regression coefficient from the fully adjusted model to that from the minimally adjusted model

Cohort	Year of birth	N	Maternal exposure	Offspring outcome	Covariates	β[a] (95% CI)	β FULL : β MINIMAL[b]
Motherwell Birth Cohort Study (13)	1967-1978	276	Pregnancy BMI (median 16 weeks gestation) (kg/m^2), calculated using height abstracted from the antenatal record and clinically measured weight	27-30 year fat mass index (kg/m^2), calculated using clinically measured weight and fat mass estimated from skinfold measurements	None	0.20 ($P < 0.001$)	1.55
					Offspring age, sex, smoking status, social class and activity level	0.31 ($P < 0.001$)	
Avon Longitudinal Study of Parents and Children (ALSPAC) (14)	1991-1992	4091	Pre-pregnancy BMI (*z*-score), calculated using self reported weight and height	9-11 year fat mass (*z*-score), from DXA scan	Offspring height, offspring height squared, paternal BMI, standardised for offspring sex, age and maternal age	0.25 (0.22, 0.27)	0.96
					As above, plus family social class, parental education, parity, parental smoking, offspring pubertal status	0.24 (0.22, 0.26)	

Note S2: Cohort sample descriptions

The Northern Finland Birth Cohort (NFBC) 1966 (15) aimed to recruit all pregnant women living in the northernmost two provinces of Finland with an expected delivery date in 1966; 96.3% of births in 1966 were included and the sample includes 12,058 live born babies. The NFBC1986 (16) aimed to recruit all pregnant women living in the same geographical area as the NFBC1966 with an expected delivery date between 1st July 1985 and 30th June 1986; 99% of births in the aforementioned time period were included, and the sample includes 9432 live born babies. Further details of the NFBCs are available at the NFBC website (17). The Avon Longitudinal Study of Parents and Children (ALSPAC) (18) enrolled 14,541 pregnant mothers who resided in the former County of Avon, United Kingdom and had an expected delivery date between April 1, 1991 and December 31, 1992; the full sample included 14,775 live born babies. The study website contains details of all the data that are available through a fully searchable data dictionary and variable search tool (19).

Note S3: Sample derivation flow charts

Flow charts are presented below for each cohort, and for the combined cohorts (**Figure S4**). For the combined cohorts the final sample size for each phenotype is not equal to the sum of the equivalent sample sizes for the individual cohorts, because the relatedness exclusion filter resulted in removal of different numbers of participants when applied to the combined cohorts as opposed to the separate cohorts. GCTA-GREML (Genomic-relatedness-based Restricted Maximum Likelihood implemented in the GCTA software) requires that cryptic (unknown) relatedness be removed to avoid confounding due to familial environment and non-additive genetic effects (20). Sensitivity analyses demonstrated that results of our primary analyses were similar at a broad range of relatedness thresholds (**Notes/Tables/Figures S23–S27**), and we found that in the NFBCs using a threshold below 0.05 resulted in exclusion of a large proportion of the sample (**Table S50**), since Finland has a relatively small founding population. Consequently, after merging data from the three cohorts we removed one individual from each cryptically related pair, using a relatedness threshold of 0.05.

Figure S4: Sample derivation flow charts

NFBC1966

N = 11,967
Babies born alive to enrolled mothers, for whom consent has not been withdrawn

→ **N = 311**
Excluded due to non-singleton birth

N = 11,656
Singletons born alive to enrolled mothers

→ **N = 6349**
Excluded due to missing genotype data

N = 5307
Singletons born alive to enrolled mothers, with genotype data available

→ **N = 0**
Siblings excluded at random (no siblings present)

N = 5307
Singletons born alive to enrolled mothers, with genotype available, excluding one of any sib-pairs present

Individuals excluded on the basis of missing phenotypic data and relatedness > 0.05:

Offspring phenotype	N with phenotypic data available[a]	N with phenotypic data available and relatedness < 0.05[b]
**Birth weight**	4711	3702
**1yr BMI**	4324	3450
**5yr BMI**	3254	2692
**10yr BMI**	3295	2706
**15yr BMI**	4654	3665
**31yr BMI**	4732	3711
**46yr BMI**	3805	3079

a. Phenotypic data defined as maternal BMI, offspring phenotype and offspring age at phenotype measurement (gestational age for birth weight)
b. Cryptically related individuals were excluded separately for each phenotype in order to maximise sample size, therefore number removed due to cryptic relatedness is slightly different for each phenotype

Figure S4 (continued): Sample derivation flow charts

NFBC1986

N = 9432
Babies born alive to enrolled mothers

N = 229
Excluded due to non-singleton birth

N = 9203
Singletons born alive to enrolled mothers

N = 5858
Excluded due to missing genotype data

N = 3345
Singletons born alive to enrolled mothers, with genotype data available

N = 0
Siblings excluded at random (no siblings present)

N = 3345
Singletons born alive to enrolled mothers, with genotype available, excluding one of any sib-pairs present

Individuals excluded on the basis of missing phenotypic data and relatedness > 0.05:

Offspring phenotype	N with phenotypic data available[a]	N with phenotypic data available and relatedness < 0.05[b]
**Birth weight**	3228	2719
**1yr BMI**	2783	2400
**5yr BMI**	2620	2265
**10yr BMI**	2563	2221
**15yr BMI**	3245	2732

a. Phenotypic data defined as maternal BMI, offspring phenotype and offspring age at phenotype measurement (gestational age for birth weight)
b. Cryptically related individuals were excluded separately for each phenotype in order to maximise sample size, therefore number removed due to cryptic relatedness is slightly different for each phenotype

Figure S4 (continued): Sample derivation flow charts

ALSPAC

N = 14,751
Babies born alive to enrolled mothers, for whom consent has not been withdrawn

N = 385
Excluded due to non-singleton birth

N = 14,366
Singletons born alive to enrolled mothers

N = 5731
Excluded due to missing genotype data

N = 8635
Singletons born alive to enrolled mothers, with genotype data available

N = 40
Siblings excluded at random (one from each sib-pair)

N = 8595
Singletons born alive to enrolled mothers, with genotype available, excluding one of any sib-pairs present

Individuals excluded on the basis of missing phenotypic data and relatedness > 0.05:

Offspring phenotype	N with phenotypic data available[a]	N with phenotypic data available and relatedness < 0.05[b]
**Birth weight**	7113	6580
**1yr BMI**	6706	6224
**5yr BMI**	6428	5991
**10yr BMI**	5899	5543
**15yr BMI**	5215	4944

a. Phenotypic data defined as maternal BMI, offspring phenotype and offspring age at phenotype measurement (gestational age for birth weight)
b. Cryptically related individuals were excluded separately for each phenotype in order to maximise sample size, therefore number removed due to cryptic relatedness is slightly different for each phenotype

Figure S4 (continued): Sample derivation flow charts

Combined cohorts (one-stage IPD meta-analysis)

N = 17247
Singletons born alive to enrolled mothers, with genotype available, excluding one individual from any sib-pairs present

Individuals excluded on the basis of missing phenotypic data and relatedness > 0.05:

Offspring phenotype	N with phenotypic data available[a]	N with phenotypic data available and relatedness < 0.05[b]
**Birth weight**	15052	11498
**1yr BMI**	13695	10733
**5yr BMI**	12394	9915
**10yr BMI**	11757	9433
**15yr BMI**	13129	9875

a. Phenotypic data defined as maternal BMI, offspring phenotype and offspring age at phenotype measurement (gestational age for birth weight)
b. Cryptically related individuals were excluded separately for each phenotype in order to maximise sample size, therefore number removed due to cryptic relatedness is slightly different for each phenotype

Note S5: Genotyping and quality control

<u>NFBC1966</u>

Genotyping was carried out at the Broad Institute using the Illumina Infinium 370cnvDuo array and the Beadstudio calling algorithm, as described previously by Sabatti *et al.* (21). Individuals were excluded due to call rate < 95%, unspecified sex, sample duplication/contamination, sex mismatch, relatedness (identity by descent [IBD]), outlying heterozygosity or withdrawal of consent, giving a sample size of 5400. Population stratification was assessed by multidimensional scaling analysis (MDS) and compared with Hapmap phase 3 reference populations; no individuals of non-European ancestry were detected. Copy number variations (CNVs) and single nucleotide polymorphisms (SNPs) with call rate < 95% (for markers with minor allele frequency [MAF] > 5%), CNVs and SNPs with call rate < 99% (for markers with MAF <5%), lack of Hardy-Weinberg equilibrium (HWE) ($P < 1.0 \times 10^{-4}$) or MAF < 1% were excluded.

<u>NFBC1986</u>

Genotyping was carried out at Imperial College London and the University of Oxford using the Illumina HumanOmniExpressExome-8v1.2 array and the GenomeStudio calling algorithm. Individuals were excluded due to call rate < 99%, sex mismatch, relatedness (reported and

identity by state [IBS]), outlying heterozygosity and non-random sampling, giving a sample size of 3384. Population stratification was assessed by MDS and compared with Hapmap phase 3 reference populations; no individuals of non-European ancestry were detected. SNPs with call rate < 99%, lack of HWE ($P < 1.0 \times 10^{-4}$) or MAF < 1% were excluded.

ALSPAC

Offspring genotyping was carried out at the Wellcome Trust Sanger Institute, Cambridge, UK and the Laboratory Corporation of America, Burlington, NC, USA using the Illumina HumanHap550 quad chip array. Individuals were excluded on the basis of gender mismatches, minimal or excessive heterozygosity, disproportionate levels of individual missingness (> 3%), and insufficient sample replication (IBD < 0.8). Population stratification was assessed by MDS and compared with Hapmap phase 2 reference populations; all individuals with non-European ancestry were removed, giving a sample size of 9115 (prior to application of the other exclusion criteria for the present study). SNPs with call rate of < 95%, lack of HWE ($P < 5.5 \times 10^{-7}$) or MAF < 1% were excluded.

Note S6: Selection of offspring anthropometric measurements within age windows

Offspring anthropometric measurements during childhood and adolescence were used at birth and at four age windows throughout childhood and adolescence, giving mean ages close to 1, 5, 10 and 15 years (target ages) (**Table S7, Table 1**). Age windows were chosen to maximise sample size and obtain measurements with a similar age distribution for all three cohorts; within windows data points were first selected to prioritise higher quality data sources (clinical exam > growth record > questionnaire) and second to minimise the age difference from the aforementioned target ages. The source of the measurements used at each age for each cohort is given in **Table S8**.

Table S7: Age windows and target ages for offspring anthropometric measurements.

Target age	Lower and upper boundaries of age window (years)
1 year	>= 0.5, < 2
5 years	>= 3, < 7
10 years	>= 8, < 12
15 years	>= 13, < 17

Table S8: Source of anthropometric measurements

Table S8: Source of anthropometric measurements used at each age by cohort

Variable	Cohort	Data source
Maternal pre-pregnancy BMI	NFBC1966	Pre-pregnancy weight reported by the mothers at the first antenatal clinic visit (average 16 weeks gestation) and height measured or self-reported at between 24 and 28 weeks gestation
	NFBC1986	Pre-pregnancy weight self-reported at 12-16 weeks gestation and height measured (53.9%) or self-reported (46.1%) between 12 and 24 weeks gestation
	ALSPAC	Height and pre-pregnancy weight reported by the mothers during pregnancy (~90% of entire baseline sample) or 4 months postnatally (~10% of entire baseline sample)
Birth weight and length	NFBC1966	Abstracted from the birth record at the time of birth
	NFBC1986	Abstracted from the birth record at the time of birth
	ALSPAC	Weight was measured by trained research assistants, abstracted form the birth record or abstracted from the birth notification. Length was measured by trained research assistants or abstracted from the birth record
1 year BMI	NFBC1966	Measured at routine child examination (97.2%) or abstracted from growth records (2.8%)
	NFBC1986	Abstracted from growth records (100%)
	ALSPAC	Measured clinically (13.2%), abstracted from growth records (85.7%) or from postal questionnaire (1.1%)
5 year BMI	NFBC1966	Abstracted from growth records (100%)
	NFBC1986	Abstracted from growth records (100%)
	ALSPAC	Measured clinically (12.4%), abstracted from growth records (75.5%) or from postal questionnaire (12.1%)
10 year BMI	NFBC1966	Abstracted from growth records (100%)
	NFBC1986	Abstracted from growth records (100%)
	ALSPAC	Measured clinically (99.1%) or from postal questionnaire (0.9%)
15 year BMI	NFBC1966	Abstracted from growth records (70.2%), or from postal questionnaire filled in by the offspring, parents or school nurse (29.8%)
	NFBC1986	Measured clinically (99.5%) or abstracted from growth records (0.5%)
	ALSPAC	Measured clinically (91.8%) or from postal questionnaire (8.2%)
31 year BMI	NFBC1966	Measured clinically (95.5%) or self-reported via postal questionnaire (4.5%)
46 year BMI	NFBC1966	Measured clinically (86.4%) or self-reported via postal questionnaire (13.6%)

Note S9: Equations for phenotypic variable standardisation and phenotypic covariance, and statistical details of the GCTA-GREML model

Phenotypic variable standardisation

We standardised phenotypic variables using the following formula: $z_i = (x_i - \bar{x})/s$, where z_i denotes the value of the standardised variable for the ith individual, x_i denotes the unstandardised variable, and $\bar{x}$ and s denote the mean and standard deviation respectively of the unstandardised variable in the combined cohorts.

Phenotypic covariance

Phenotypic covariance between maternal BMI and offspring phenotype was calculated using the usual formula:

$$\mathrm{cov}(x, y) = \frac{1}{N-1}\sum_{i=1}^{N}(x_i - \bar{x})(y_i - \bar{y}), \qquad \text{Equation 1}$$

where x_i and y_i are the values of maternal BMI and offspring phenotype respectively for the ith mother-child dyad, and $\bar{x}$ and $\bar{y}$ are the corresponding means.

GCTA-GREML

For our primary analyses we used the bivariate GCTA-GREML model to partition the phenotypic covariance between maternal BMI and offspring phenotype (Cov_P) into that explained by additive genetic effects captured by offspring SNPs (referred to as the genetic covariance [Cov_G]) and residual (unexplained) covariance (Cov_E). We then calculated the ratio Cov_G:Cov_P, which has been referred to as the bivariate heritability (22). For clarity however it is helpful to first outline the univariate GCTA-GREML model, which partitions the phenotypic variance of a single trait (Var_P) into that explained by additive genetic effects captured by SNPs (referred to as the genetic variance [Var_G]), and residual variance (Var_E), in order to estimate the SNP-based heritability of the phenotype (Var_G/Var_P). Specifically, this is the narrow-sense heritability (denoted h^2), because the numerator includes only the phenotypic variance explained by additive genetic effects. **Note S13** and **Table S14** enable comparison of our primary bivariate GCTA-GREML results to univariate GCTA-GREML results from the same sample.

Univariate and bivariate GCTA-GREML have been described in full in the original methods papers (23, 24); here we adapt the notation and description of the models from Deary *et al.* (25).

In univariate GCTA-GREML the variance of the phenotype is partitioned using a linear mixed model:

$$y_i = \beta_0 + \beta_1 x_i + g_i + e_i \qquad \text{Equation 2}$$

where y_i is the phenotypic value, β_0 is the intercept, β_1 and x_i are the coefficient and indicator variable respectively for a covariate modelled as a fixed effect, g_i is the additive genetic value (the sum of the additive effects of all SNPs) for the ith individual, modelled as a random effect with $g_i \sim N(0, \sigma_g^2)$ and e_i is the residual with $e_i \sim N(0, \sigma_e^2)$. Under this model the phenotypic covariance between pairs of distantly related individuals is modelled as a function of their genetic similarity:

$$cov(y_i, y_j) = A_{ij}\sigma_g^2, \text{ and} \qquad \text{Equation 3}$$

$$var(y_i) = A_{ii}\sigma_g^2 + \sigma_e^2, \qquad \text{Equation 4}$$

where A_{ij} is the genetic relationship between individuals i and j estimated from the SNP data.

Equation 1 can be re-written in matrix form as:

$$\mathbf{y} = \mathbf{Xb} + \mathbf{g} + \mathbf{e}, \qquad \text{Equation 5}$$

where $\mathbf{y} = \{y_i\}_{n\times1}$ is a vector of phenotypic values with n being the sample size,

$\mathbf{b} = \{\beta_q\}_{(Q+1)\times1}$ is a vector of fixed effects (with Q being the number of fixed effects), $\mathbf{X} = \{X_i\}_{n\times(Q+1)}$ is an incidence matrix for the fixed effects, $\mathbf{g} = \{g_i\}_{n\times1}$ is a vector of additive genetic values and $\mathbf{e} = \{e_i\}_{n\times1}$ is a vector of residuals. The variance-covariance matrix is then:

$$var(\mathbf{y}) = \mathbf{V} = \mathbf{A}\sigma_g^2 + \mathbf{I}\sigma_e^2, \qquad \text{Equation 6}$$

where $\mathbf{A} = \{\boldsymbol{A_{ij}}\}_{\boldsymbol{n\times n}}$ is the genetic relatedness matrix (GRM) and $\mathbf{I}$ is an $\boldsymbol{n} \times \boldsymbol{n}$ identity matrix. It has been shown previously (23) that the model specified by Equation 4 is equivalent to a model fitting the effects of all the SNPs together:

$$\mathbf{y} = \mathbf{Xb} + \mathbf{Wu} + \mathbf{e}, \qquad \text{Equation 7}$$

where $\mathbf{W} = \{w_{im}\}_{n\times M}$ is an incidence matrix for the M SNPs;

$$w_{im} = (x_{im} - 2p_m)/\sqrt{2p_m(1-p_m)}, \qquad \text{Equation 8}$$

with x_{im} denoting the genotype for the ith individual at the mth SNP, coded 0, 1 or 2 corresponding to genotypes *bb*, *Bb* and *BB*, and p_m denotes the allele frequency of the *B* allele at locus m; $\mathbf{u}$ is an $M \times 1$ vector of SNP effects, $\mathbf{u}{\sim}N(\mathbf{0}, \mathbf{I}\sigma_u^2)$. These SNP effects are assumed to be uncorrelated because the model is equivalent to fitting all SNPs jointly (20). **Equation 8** standardises the genotypes such that they have mean zero and unit variance. We define

$$\mathbf{A} = \mathbf{WW}'/M. \qquad \text{Equation 9}$$

The mean A_{ij} (GRM off-diagonals) is zero, and in the absence of inbreeding the mean A_{ii} (GRM diagonals) is unity. Since the additive genetic value is the sum of the SNP effects,

$$\sigma_g^2 = M\sigma_u^2. \qquad \text{Equation 10}$$

The variance-covariance matrix will then be:

$$\mathbf{V} = \mathbf{WW}'\sigma_u^2 + \mathbf{I}\sigma_e^2 = \mathbf{A}\sigma_g^2 + \mathbf{I}\sigma_e^2. \qquad \text{Equation 11}$$

We fitted this model using the GCTA software, which implements the average information (AI) algorithm (26) to give restricted maximum likelihood (REML) estimates of σ_g^2 and σ_e^2.

GCTA-GREML can be extended to a bivariate model (24) to partition the covariance of two phenotypes:

$\mathbf{y}_1 = \mathbf{X}_1\mathbf{b}_1 + \mathbf{g}_1 + \mathbf{e}_1$ for trait one (maternal BMI), and Equation 12

$\mathbf{y}_2 = \mathbf{X}_2\mathbf{b}_2 + \mathbf{g}_2 + \mathbf{e}_2$ for trait two (offspring phenotype), Equation 13

with $\mathbf{g}_1{\sim}N(\mathbf{0}, \mathbf{A}\sigma_{g1}^2)$, $\mathbf{e}_1{\sim}N(\mathbf{0}, \mathbf{I}\sigma_{e1}^2)$, the distributions of $\mathbf{g}_2$ and $\mathbf{e}_2$ specified similarly, and notation as for **Equation 5** above with the subscripts “1” and “2” denoting the two traits. The phenotypic

covariance between pairs of distantly related individuals for trait one, trait two and between traits one and two is then modelled as a function of their genetic similarity:

$$cov(y_{i1}, y_{j1}) = A_{ij}\sigma_{g1}^2, \quad \text{Equation 14}$$

$$var(y_{i1}) = A_{ii}\sigma_{g1}^2 + \sigma_{e1}^2, \quad \text{Equation 15}$$

$$cov(y_{i2}, y_{j2}) = A_{ij}\sigma_{g2}^2, \quad \text{Equation 16}$$

$$var(y_{i2}) = A_{ii}\sigma_{g2}^2 + \sigma_{e2}^2, \quad \text{Equation 17}$$

$$cov(y_{i1}, y_{j2}) = A_{ij}\sigma_{g1g2}, \quad \text{Equation 18}$$

$$cov(y_{i1}, y_{i2}) = A_{ii}\sigma_{g1g2} + \sigma_{e1e2}, \quad \text{Equation 19}$$

where σ_{g1g2} and σ_{e1e2} denote the genetic and residual covariance between the two traits respectively. In matrix notation the variance-covariance matrix can be written as:

$$\text{var}\begin{bmatrix}\mathbf{y}_1\\ \mathbf{y}_2\end{bmatrix} = \begin{bmatrix}\mathbf{A}\sigma_{g1}^2 + \mathbf{I}\sigma_{e1}^2 & \mathbf{A}\sigma_{g1g2} + \mathbf{I}\sigma_{e1e2}\\ \mathbf{A}\sigma_{g1g2} + \mathbf{I}\sigma_{e1e2} & \mathbf{A}\sigma_{g2}^2 + \mathbf{I}\sigma_{e2}^2\end{bmatrix}. \quad \text{Equation 20}$$

We fitted this bivariate model using the GCTA software (26), to obtain REML estimates of variance and covariance components.

Note S10: Sensitivity analysis 1—Phenotypic variable transformations

We investigated whether Cov_G:Cov_P estimates were sensitive to the transformation used to normalise skewed variables (all phenotypic variables aside from birth weight (BW) were positively skewed). Four transformations were investigated:

1. Standardisation only (i.e. no normalising transformation)
2. Natural log followed by standardisation
3. Rank based inverse normal transformation
4. *Z*-score calculated using the UK-WHO Child Growth Reference (for weight at birth [standardised for sex and gestational age], and for BMI from age 1 to 15 years [standardised for sex and age at BMI measurement])

Figure S11 shows that normalising transformations made little difference to the Cov_G:Cov_P estimates; we have therefore presented the analyses with no normalising transformation as the primary results.

Figure S11: Cov_G:Cov_P estimates for maternal BMI and offspring phenotype, varying the phenotypic transformation used for skewed phenotypic variables, in the combined cohorts (pooled IPD estimates). Transformations: **1.** Standardisation only (i.e. no normalising transformation) **2.** Natural log followed by standardisation **3.** Rank based inverse normal transformation **4.** *Z*-score calculated using the UK-WHO Child Growth Reference (for weight at birth [standardised for sex and gestational age], and for BMI from age 1 to 15 years [standardised for sex and age at BMI measurement])

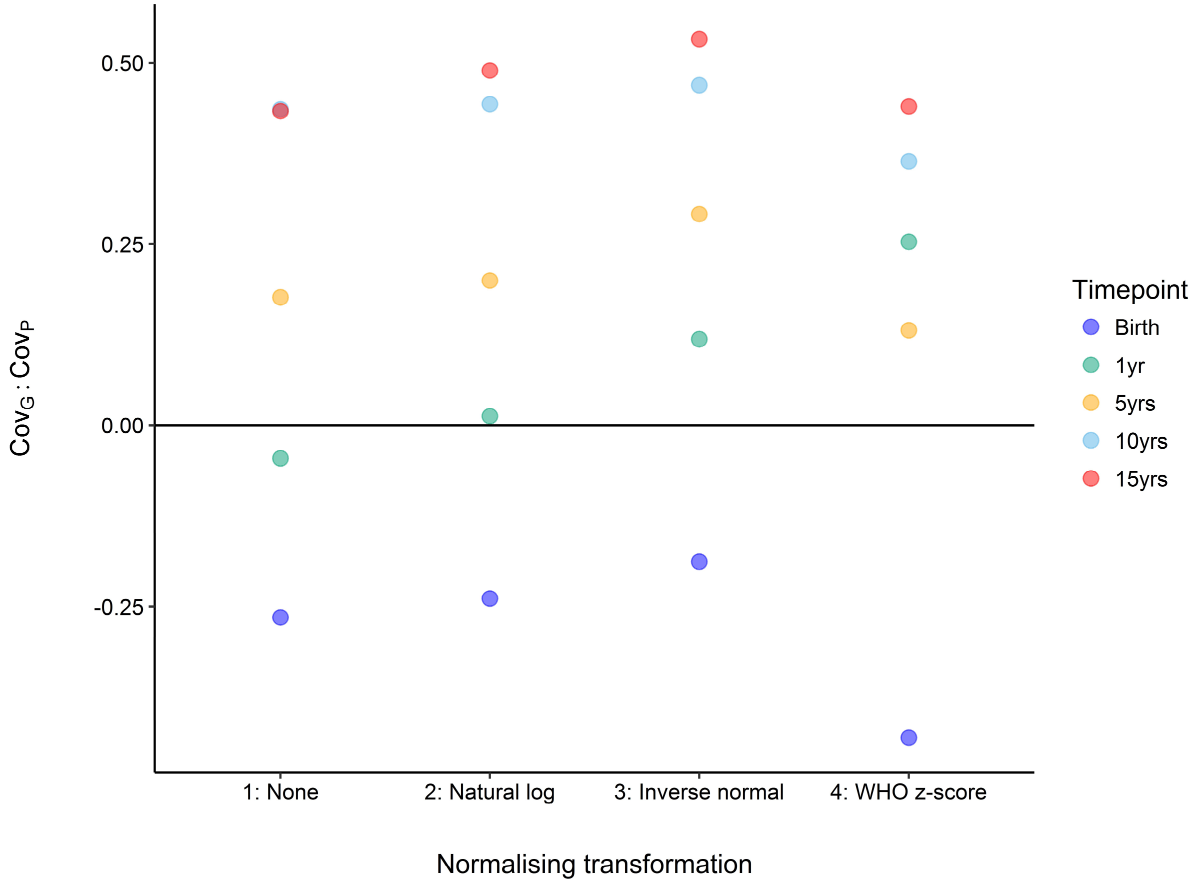

Note S12: Source of other variables

As part of Sensitivity analysis 3 (**Notes/Tables/Figures S20–S24**) we included potential confounders of the maternal BMI-offspring BMI association as fixed effects whilst fitting the bivariate GCTA-GREML model. Details of the variables used in this analysis are given immediately below.

<u>NFBC1966</u>

Questionnaires were filled in by mothers during pregnancy between 24 and 28 weeks gestation and trained midwives recorded prospective data on the pregnancies on a structured study form from the 16th gestational week onwards using the maternity records. Gestational age and maternal age at delivery were from the maternity records; gestational age was calculated based

on the last menstrual period. Maternal age at delivery was calculated from offspring date of delivery and maternal date of birth. Maternal parity, smoking and socioeconomic position (SEP) variables were derived using data from the pregnancy questionnaire; parity was defined as the number of previous pregnancies resulting in live or stillbirth. Using data on smoking behaviour in the second month of pregnancy a maternal smoking variable was derived with the following categories: non-smoking, light to moderate smoking (10 or less cigarettes per day) or heavy smoking (greater than 10 cigarettes per day). Socioeconomic position (SEP) in pregnancy was defined based on paternal occupation and grouped into the following categories: class I (professionals), class II (professionals, lower), class III (skilled workers), class IV (unskilled workers and no occupation), farmers with land >= 8 hectares and farmers with land < 8 hectares.

NFBC1986

Questionnaires were filled in by the mothers during early pregnancy (at 12-16 weeks gestation) and late pregnancy (from 24 weeks gestation onwards). Gestational age was recorded on a structured study form by the midwives attending the birth, and was calculated from early pregnancy ultrasound scan or the date of the last menstrual period. Maternal age at delivery was calculated from maternal date of birth and offspring date of delivery; maternal parity data were from the early pregnancy questionnaire. Maternal SEP data were from the late pregnancy questionnaire; SEP during pregnancy was defined based on paternal occupation and grouped into the following categories: professional/entrepreneur, skilled worker (non-manual), skilled worker (manual), unskilled worker/apprentice, farmer, student/at home and sick pension/unemployed. Using questionnaire data on smoking behaviour after the second month of pregnancy a maternal smoking variable was derived with the following categories: non-smoking, light to moderate smoking (10 or less cigarettes per day) or heavy smoking (greater than 10 cigarettes per day).

ALSPAC

Parity was reported by the mothers in a questionnaire completed at 18 weeks gestation. SEP during pregnancy was derived from data on maternal and paternal occupation reported by the mother in a questionnaire completed at 32 weeks gestation; the highest occupational group of the mother or father was used to give a variable with the following categories: class I (professional occupations), class II (managerial and technical occupations), class III (skilled manual occupations), class IV (partly skilled occupations) and class V (unskilled occupations). Data on maternal smoking during pregnancy were from questionnaires completed by the mother at 18 and 32 weeks gestation; a variable was created with three categories: never smoked during pregnancy, smoked in early pregnancy only and smoked throughout pregnancy. Maternal age at delivery was calculated from maternal date of birth and offspring date of delivery. Data on gestational age at delivery were obtained from birth records, and consistent with UK clinical practice at the time will have largely been based on the date of the mothers' last menstrual period, with some potential modification in those who had an ultrasound scan in the first trimester or on clinical assessment at birth.

Note S13: Univariate GCTA-GREML estimates of SNP heritability

For our primary analyses, we used bivariate GCTA-GREML to estimate SNP-based bivariate heritability (Cov_G:Cov_P) for maternal BMI and offspring phenotypes, as explained in the **Main Text** and **Note S9**. It is also possible to fit a univariate GCTA-GREML model, in order to estimate SNP-based univariate heritability (i.e. Var_G/Var_P, where Var_G is the phenotypic variance explained by the additive genetic effects captured by imputed SNPs, and Var_P is the phenotypic variance). Estimates of SNP-based univariate heritability (denoted h^2_{SNP}) are presented below for the same offspring phenotypes that we investigated in our primary analyses. Comparison of the estimates from **Table S14** with our main results (**Figure 2** in **Main Text**) illustrates the difference between bivariate SNP heritability and univariate SNP heritability. If univariate SNP heritability for maternal and offspring phenotypes is substantial, and the maternal and offspring phenotypes are genetically correlated (**Note S15**), this would suggest that the covariance between maternal and offspring phenotypes is due in part to genetic effects. However, our bivariate SNP heritability results go further than this by empirically quantifying the extent to which the maternal-offspring phenotypic covariance is explained by genetic effects captured by SNPs; they also enable comparison of bivariate heritability for different offspring phenotypes (e.g. birth weight versus adolescent BMI).

Table S14: Univariate GCTA-GREML estimates of $\boldsymbol{h^2_{\mathrm{SNP}}}$, from the combined cohorts. 95% confidence intervals were calculated as the point estimate +/- 1.96 x SE_{GCTA}, where SE_{GCTA} denotes the standard error as supplied by the GCTA software. Phenotypes were treated as for the primary bivariate GCTA-GREML analyses, 20 principal components, cohort, offspring sex and age at phenotype measurement (replaced with gestational age at birth for BW models) were included as fixed effects, a relatedness exclusion threshold of 0.05 was applied, and the "--reml-no-constrain" option was used

Offspring phenotype	h^2_{SNP}		N
	**Point estimate**	**95% CI**	
Birth weight	0.27	0.21, 0.33	12 438
1 year BMI	0.34	0.27, 0.41	11 555
5 year BMI	0.26	0.19, 0.33	10 732
10 year BMI	0.35	0.28, 0.42	10 381
15 year BMI	0.27	0.20, 0.34	10 719

Note S15: Relationship of bivariate heritability to genetic correlation

Genetic correlation (r_G) is defined as the correlation between the additive genetic values for two traits (with additive genetic value defined as the sum the additive effects of an individual's alleles). r_G is a measure of the extent to which the additive genetic effects are shared by the two phenotypes (with the caveat that r_G may also be due to assortative mating or indirect genetic effects), and is calculated by the equation:

$$r_{\mathrm{G}} = \frac{Cov_{\mathrm{G}}}{\sqrt{Var_{\mathrm{G1}} \times Var_{\mathrm{G2}}}}, \qquad \text{(Equation 21)}$$

where Cov_{G} and Var_{G} are the parts of the phenotypic covariance and variance respectively which are explained by additive genetic effects, and the subscripts "1" and "2" denote phenotypes one and two.

Bivariate heritability (Cov_G:Cov_P) is a measure of the extent to which the total phenotypic covariance is explained by additive genetic effects, and is a function of r_G, the genetic variance of both traits, and the phenotypic covariance.

Note S16: Sensitivity analysis 2—SNPs used to calculate the genetic relatedness matrix

For our primary analyses we used SNPs with minor allele frequency (MAF) > 1%, imputation r^2 > 0.3 and Hardy-Weinberg equilibrium (HWE) *P* value > 1e-6. We investigated whether SNP heritability (i.e. the proportion of phenotypic variance explained by additive genetic effects captured by genome-wide imputed SNPs [h^2_{SNP}]) and Cov_G:Cov_P estimates were sensitive to the MAF and imputation r^2 thresholds applied to select the SNPs from which the genetic relatedness matrix (GRM) was calculated. Four sets of SNPs were used (for all sets a HWE *P* value threshold of 1e-6 was also applied):

1. Common imputed SNPs: MAF > 1%, imputation r^2 > 0.3
2. High quality imputed SNPs: minor allele count (MAC) > 5, imputation r^2 > 0.99
3. Array SNPs: only SNPs that were directly genotyped on the genome-wide microarray
4. All imputed SNPs with MAC > 5, imputation r^2 > 0.3

We fitted models separately for the individual cohorts and compared weighted mean estimates across all cohorts and phenotypes (weighted by sample size). **Table S17** and **Figures S18** and **S19** show that mean estimated h^2_{SNP} was somewhat higher when using all SNPs with MAC > 5 and r^2 > 0.3 (consistent with previously published findings(27)), and somewhat lower when using high quality imputed SNPs, than when using common imputed SNPs or array SNPs. However, in bivariate analyses Cov_G:Cov_P estimates did not differ markedly when using high quality imputed SNPs versus common imputed SNPs, although Cov_G:Cov_P estimates were somewhat higher when using all SNPs with MAC > 5 and r^2 > 0.3. These results provide reassurance that the estimates from our primary analyses (using SNPs with MAF > 1% and r^2 > 0.3) would not have been markedly different had we used only high quality imputed SNPs, and are conservative relative to estimates using all SNPs with MAC > 5 and r^2 > 0.3.

Table S17: Weighted mean h^2_{SNP} and Cov_G:Cov_P estimates across cohorts and phenotypes (weighted by sample size), for different SNP sets. †: The absolute value of a number is its non-negative value without regard to its sign

SNPs	Weighted mean of h^2_{SNP} estimates	Weighted mean of Cov_G:Cov_P estimates	Weighted mean of absolute values† of Cov_G:Cov_P estimates
1: MAF>0.01, r^2>0.3	0.323	0.271	0.530
2: MAC>5, r^2>0.99	0.257	0.247	0.465
3: Array	0.312	0.301	0.527
4: MAC>5, r^2>0.3	0.376	0.345	0.667

Figure S18: SNP heritability estimates at varying SNP minor allele frequency and imputation quality thresholds. **Black crosses** represent the weighted mean of the estimates across cohorts and phenotypes (weighted by sample size)

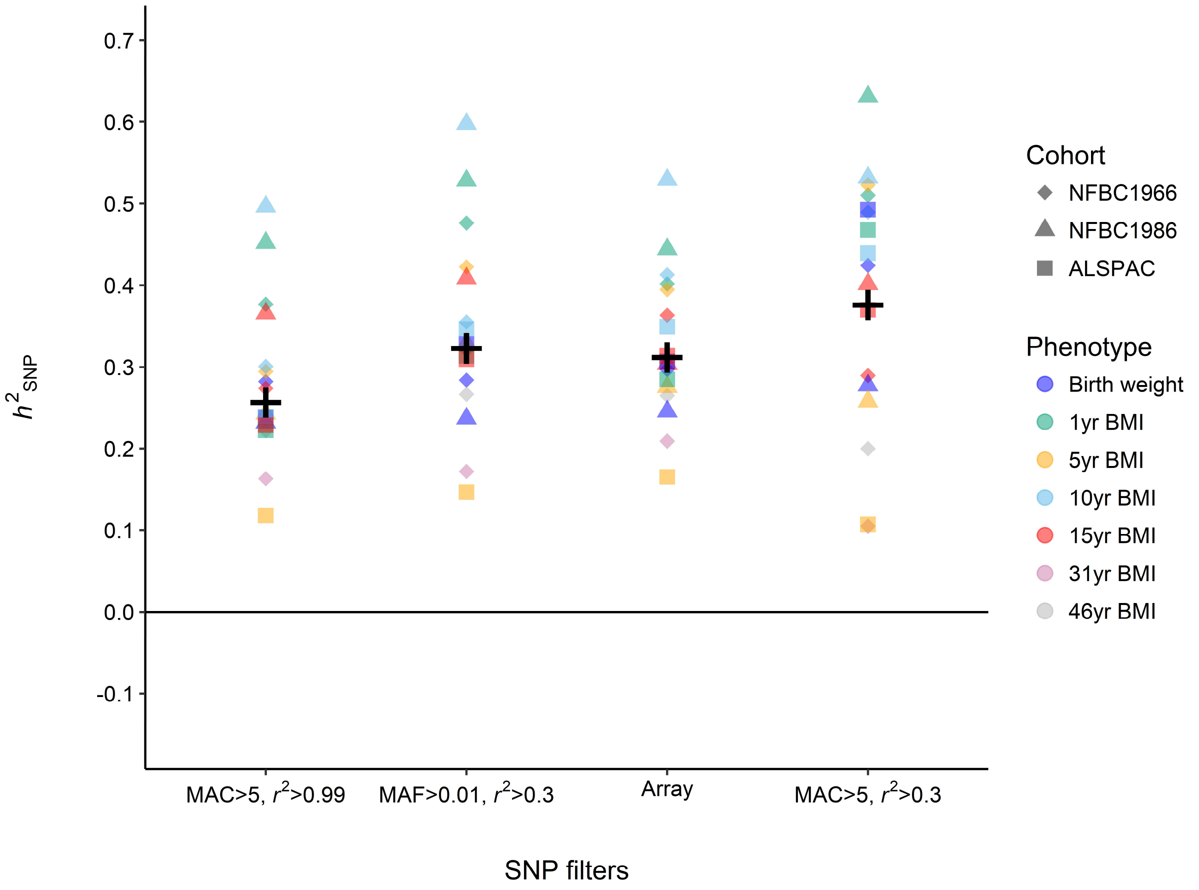

Figure S19: Cov_G:Cov_P estimates for maternal BMI and offspring phenotype, at varying SNP minor allele frequency and imputation quality thresholds. **Black crosses** represent the weighted mean (weighted by sample size) of the estimates across cohorts and phenotypes, **black stars** represent the weighted mean of the absolute values of the estimates across cohorts and phenotypes. The absolute value of a number is its non-negative value without regard to its sign

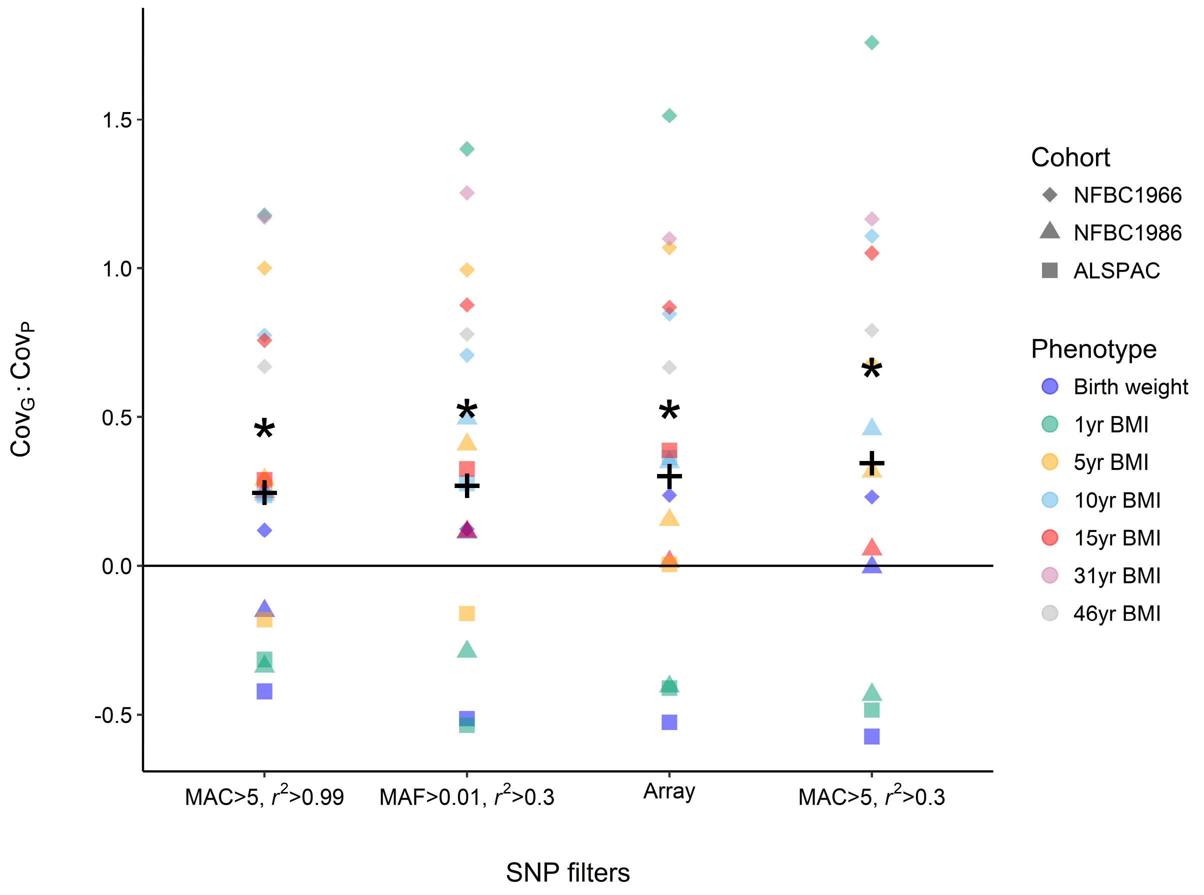

Note S20: Sensitivity analysis 3—Number of principal components and other covariates

We investigated whether h^2_{SNP} and $\mathrm{Cov_G{:}Cov_P}$ estimates varied as the number of ancestry informative principal components (PCs) that were fitted as fixed effects was varied between zero and one thousand. **Figures/Tables S21–23** show that there was a slight upward trend in h^2_{SNP} estimates as the number of PCs was increased from zero to one hundred, beyond which estimates became somewhat unstable. A gradual downward trend in h^2_{SNP} estimates as more PCs are fitted might indicate inflation of h^2_{SNP} estimates due to residual population stratification uncontrolled by PCs (28). We saw no such downward trend, providing reassurance that our results are not substantively biased by population stratification.

For the pooled IPD estimates we also investigated whether including different covariates as fixed effects impacted our h^2_{SNP} and $\mathrm{Cov_G{:}Cov_P}$ estimates. We fitted three models:

1. No covariates
2. Minimally adjusted: offspring sex and age (for childhood BMI) or gestational age (for BW)
3. Fully adjusted: as per the minimally adjusted model, plus adjustment for potential confounders of the maternal BMI-offspring phenotype association: maternal age at offspring birth, maternal smoking during pregnancy, socioeconomic position and parity at the time of pregnancy of the index offspring

Estimates for h^2_{SNP} and $\mathrm{Cov_G{:}Cov_P}$ were broadly similar across models (results available on request).

Figure S21: SNP heritability estimates with varying numbers of principal components fitted as fixed effects, in the combined cohorts (pooled IPD estimates). **Black crosses** represent the weighted mean of the estimates across phenotypes (weighted by sample size).

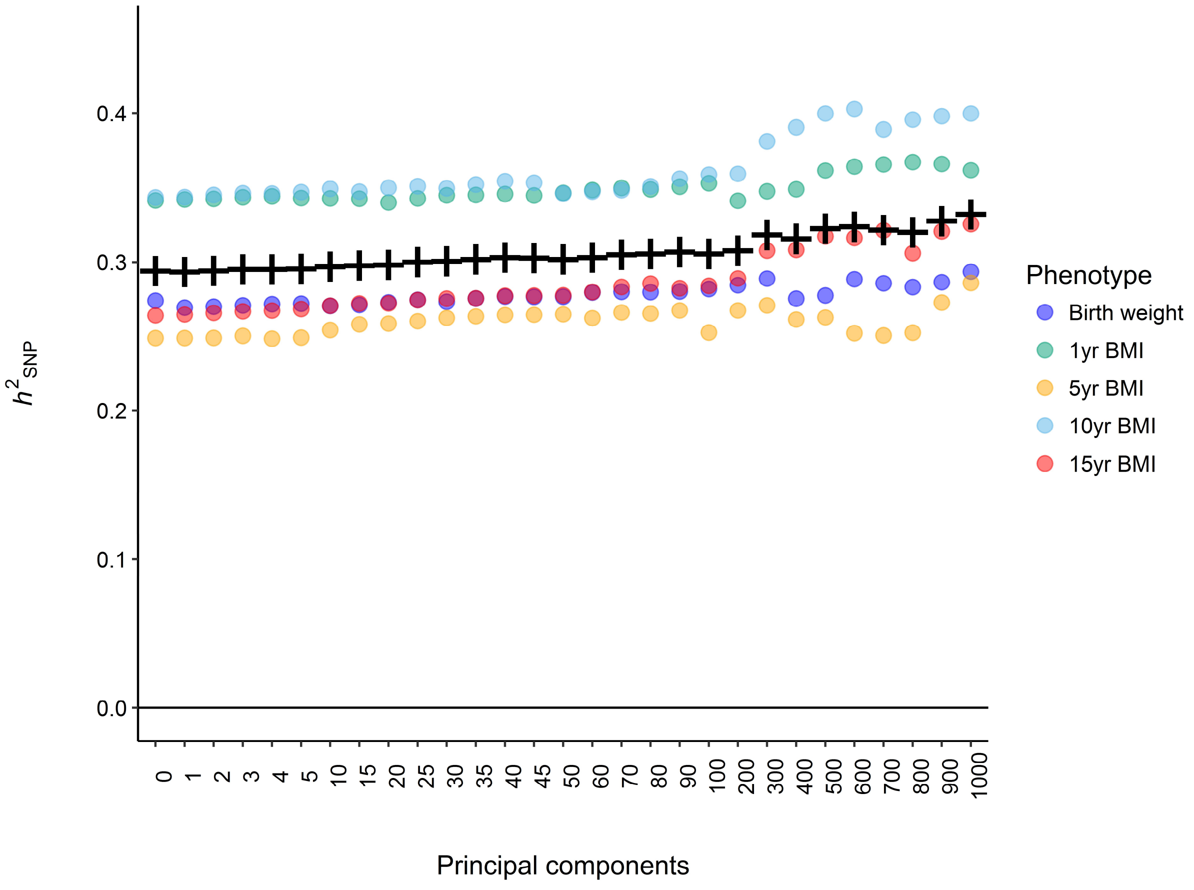

Table S22: Weighted mean SNP heritability estimates with varying numbers of principal components fitted as fixed effects, for all phenotypes in the combined cohorts (pooled IPD estimates), weighted by sample size

Number of PCs	Weighted mean of h^2_{SNP} estimates	Number of PCs	Weighted mean of h^2_{SNP} estimates
0	0.294	60	0.303
1	0.293	70	0.305
2	0.294	80	0.306
3	0.295	90	0.307
4	0.295	100	0.306
5	0.296	200	0.308
10	0.297	300	0.318
15	0.298	400	0.316
20	0.298	500	0.322
25	0.300	600	0.324
30	0.301	700	0.322
35	0.302	800	0.320
40	0.303	900	0.328
45	0.303	1000	0.332
50	0.302		

Figure S23: Cov_G:Cov_P estimates for maternal BMI and offspring phenotype, with varying numbers of principal components fitted as fixed effects, in the combined cohorts (pooled IPD estimates). **Black crosses** represent the weighted mean (weighted by sample size) of the estimates across phenotypes, **black stars** represent the weighted mean of the absolute values of the estimates across phenotypes. The absolute value of a number is its non-negative value without regard to its sign

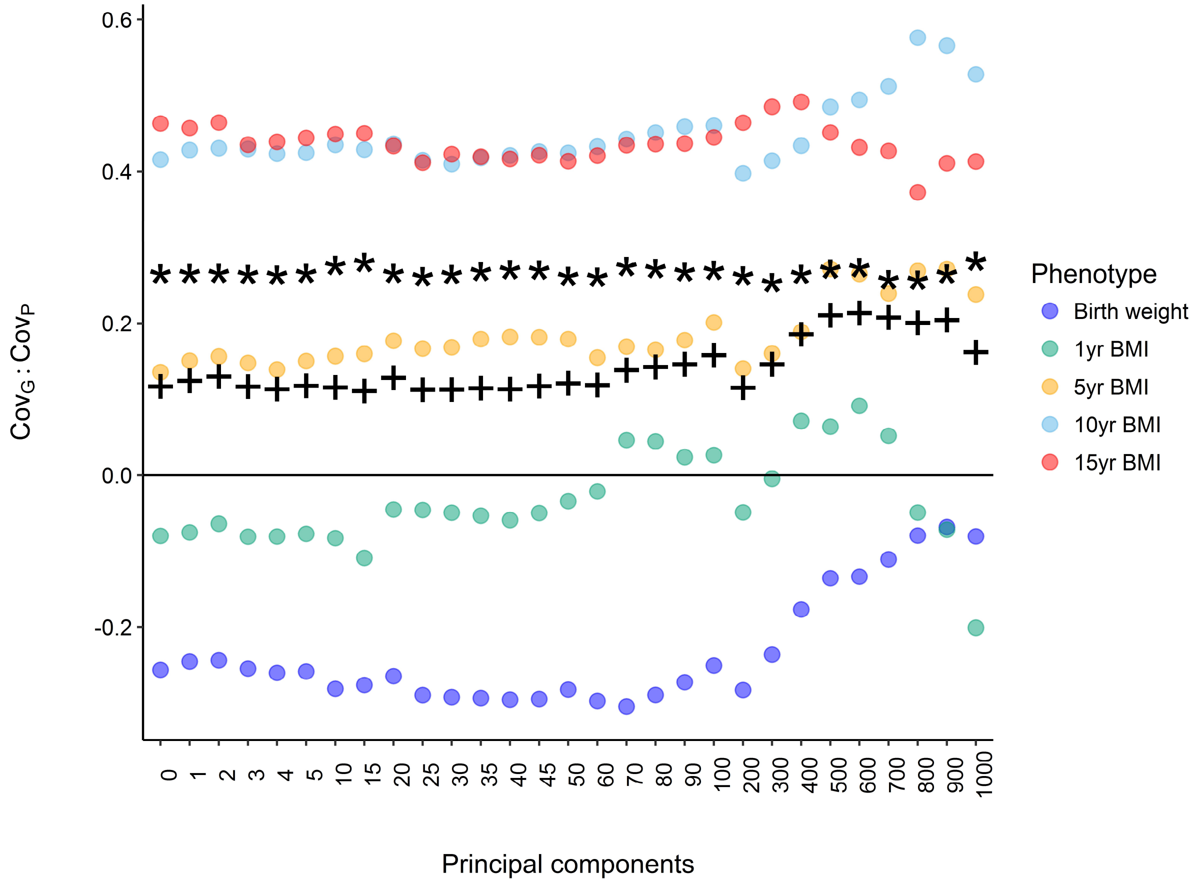

Table S24: Weighted mean Cov_G:Cov_P estimates for maternal BMI and offspring phenotype with varying numbers of principal components fitted as fixed effects, for all phenotypes in the combined cohorts (pooled IPD estimates), weighted by sample size. **†:** The absolute value of a number is its non-negative value without regard to its sign

Number of PCs	Weighted mean of Cov_G:Cov_P estimates	Weighted mean of absolute values† of Cov_G:Cov_P estimates	Number of PCs	Weighted mean of Cov_G:Cov_P estimates	Weighted mean of absolute values† of Cov_G:Cov_P estimates
0	0.117	0.265	60	0.119	0.261
1	0.125	0.266	70	0.139	0.275
2	0.130	0.266	80	0.143	0.272
3	0.117	0.264	90	0.146	0.268
4	0.114	0.264	100	0.158	0.27
5	0.118	0.266	200	0.116	0.263
10	0.116	0.276	300	0.146	0.254
15	0.111	0.280	400	0.186	0.265
20	0.129	0.266	500	0.211	0.271
25	0.113	0.261	600	0.214	0.273
30	0.113	0.264	700	0.208	0.257
35	0.115	0.268	800	0.201	0.257
40	0.114	0.271	900	0.205	0.265
45	0.118	0.270	1000	0.162	0.282
50	0.121	0.262			

Note S25: Sensitivity analysis 4—Relatedness exclusion threshold

We investigated whether estimates of h^2_{SNP} and Cov_G:Cov_P were sensitive to the relatedness threshold used to exclude cryptically related individuals from the sample. If estimates increase on relaxation of the relatedness threshold this could suggest that estimates at less stringent thresholds are biased upwards due to confounding of the familial environment with genetic similarity. **Figure S26** and **Table S27** show that there was a slight upward trend in h^2_{SNP} estimates as the relatedness threshold was relaxed from 0.025 towards 0.2 (i.e. to allow more closely related individuals to remain in the sample). **Figure S28** and **Table S29** show that the weighted mean of the Cov_G:Cov_P estimates was somewhat lower at relatedness threshold 0.025 compared to less stringent thresholds, and that this was partly driven by more extreme negative Cov_G:Cov_P estimates, such that the weighted mean of the absolute values of Cov_G:Cov_P was similar at all thresholds. We conclude that

1. It is possible that there is a small amount of inflation of our Cov_G:Cov_P and h^2_{SNP} estimates due to cryptic relatedness
2. Because of the markedly lower sample size at relatedness threshold 0.025 it is difficult to distinguish any true inflation effects from chance differences due to sampling error. The absence of a smooth upward trend in estimates on relaxation of the relatedness threshold for most phenotypes suggests that sampling error is an important cause of differences in estimates between relatedness thresholds

3. Taken together with the results of a test which uses genome partitioning and showed no evidence of h^2_{SNP} inflation due to cryptic relatedness or population stratification at relatedness threshold 0.05 (**Note S34** and **Table S35**), these results provide reassurance that any inflation of our estimates due to cryptic relatedness, if present, is likely to be minor.

Figure S26: SNP heritability estimates at varying relatedness thresholds for all phenotypes, in the combined cohorts (pooled IPD estimates). **Black crosses** represent the weighted mean of the estimates across phenotypes (weighted by sample size).

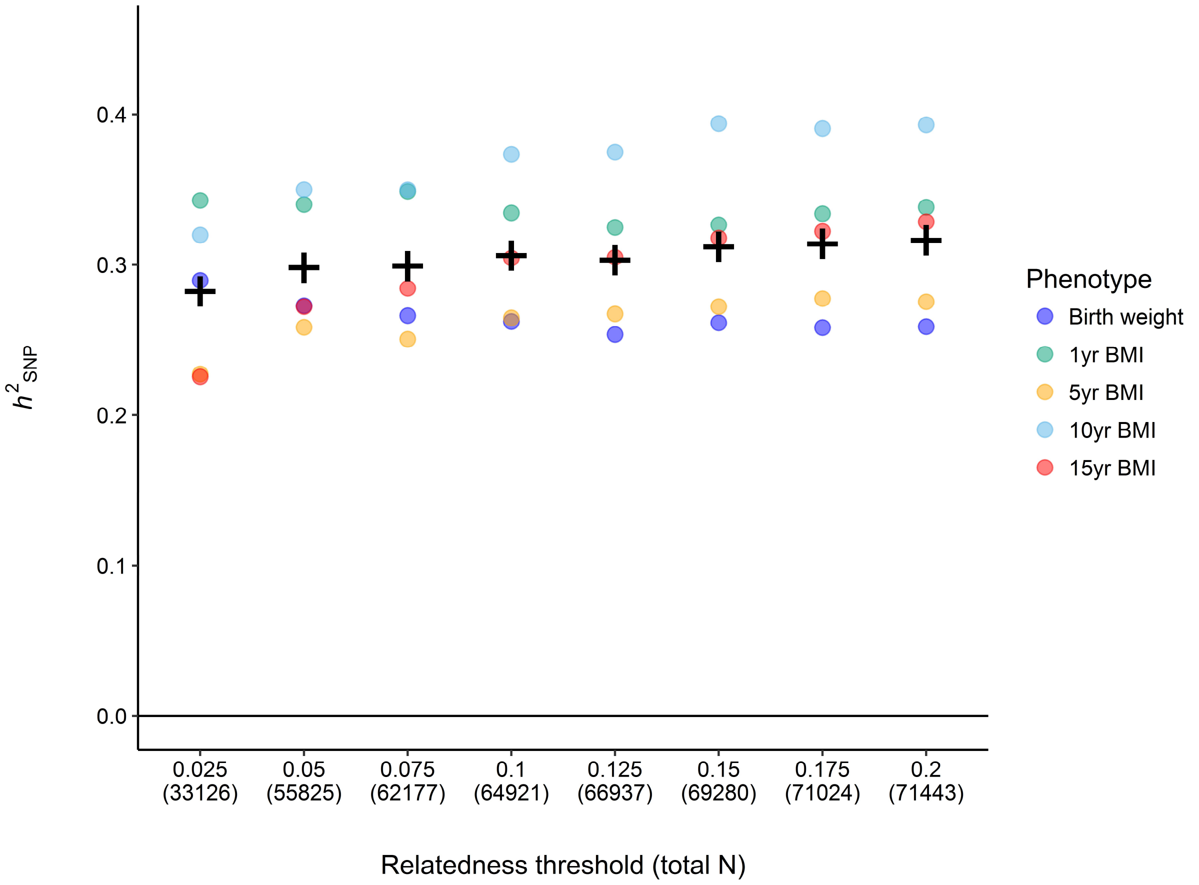

Table S27: Weighted mean SNP heritability estimates at varying relatedness thresholds for all phenotypes, in the combined cohorts (pooled IPD estimates), weighted by sample size

Relatedness threshold	N	Weighted mean of h^2_{SNP} estimates
0.025	33126	0.282
0.05	55825	0.298
0.075	62177	0.299
0.1	64921	0.306
0.125	66937	0.303
0.15	69280	0.312
0.175	71024	0.314
0.2	71443	0.316

Figure S28: Cov_G:Cov_P estimates for maternal BMI and offspring phenotype, at varying relatedness thresholds, in the combined cohorts (pooled IPD estimates). **Black crosses** represent the weighted mean (weighted by sample size) of the estimates across phenotypes, **black stars** represent the weighted mean of the absolute values of the estimates across phenotypes. The absolute value of a number is its non-negative value without regard to its sign

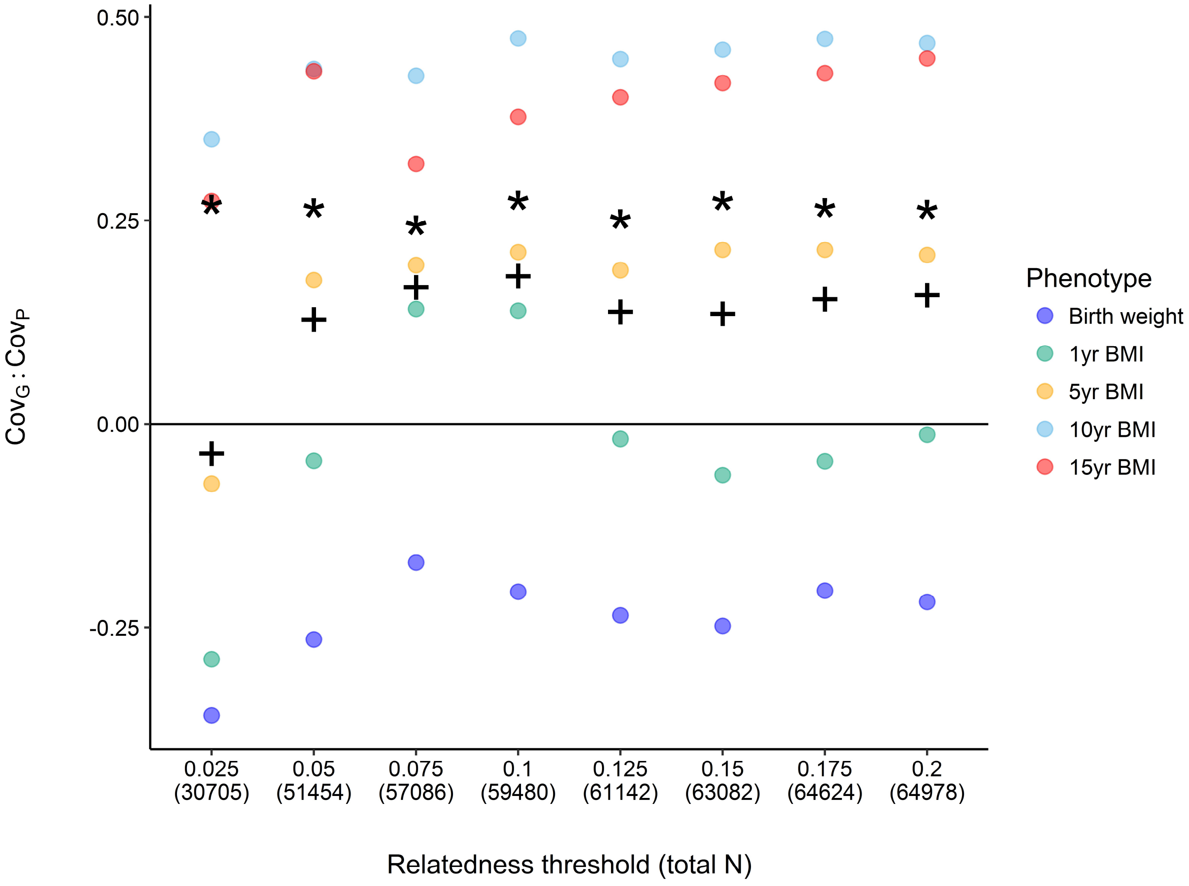

Table S29: Weighted mean Cov_G:Cov_P estimates for maternal BMI and offspring phenotype at varying relatedness thresholds, for all phenotypes in the combined cohorts (pooled IPD estimates), weighted by sample size. **†:** The absolute value of a number is its non-negative value without regard to its sign

Combined N (all phenotypes)	Relatedness threshold	Weighted mean of Cov_G:Cov_P estimates	Weighted mean of absolute values† of Cov_G:Cov_P estimates
30705	0.025	-0.036	0.27
51454	0.05	0.129	0.266
57086	0.075	0.168	0.245
59480	0.1	0.182	0.275
61142	0.125	0.138	0.252
63082	0.15	0.136	0.275
64624	0.175	0.154	0.266
64978	0.2	0.159	0.264

Note S30: Sensitivity analysis 5—Alternative phenotypes

We investigated whether Cov_G:Cov_P estimates for maternal BMI and offspring phenotype varied when replacing offspring phenotype at all ages with weight, BMI and ponderal index (PI [weight {kg} / height {m}^{3}]). **Figures S31–S33** show that there were not large differences in Cov_G:Cov_P for weight, BMI and PI. Importantly, our main conclusions would have changed little had we used BMI or PI as the primary phenotype at birth instead of BW.

Figure S31: Partitioned covariance between maternal BMI and offspring weight at all ages in the combined cohorts (pooled IPD estimates). **W**: weight at the indicated age (years)

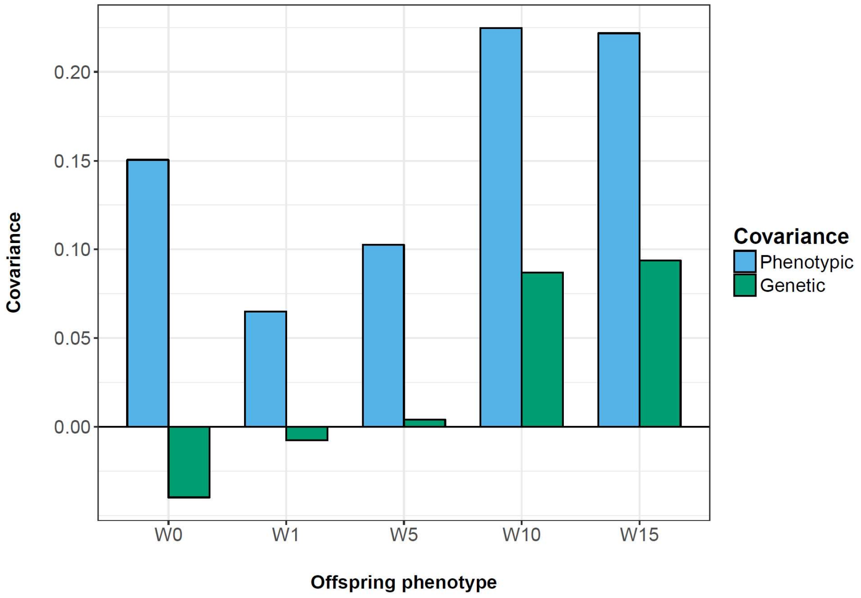

Figure S32: Partitioned covariance between maternal BMI and offspring BMI at all ages in the combined cohorts (pooled IPD estimates). **BMI**: body mass index at the indicated age (years)

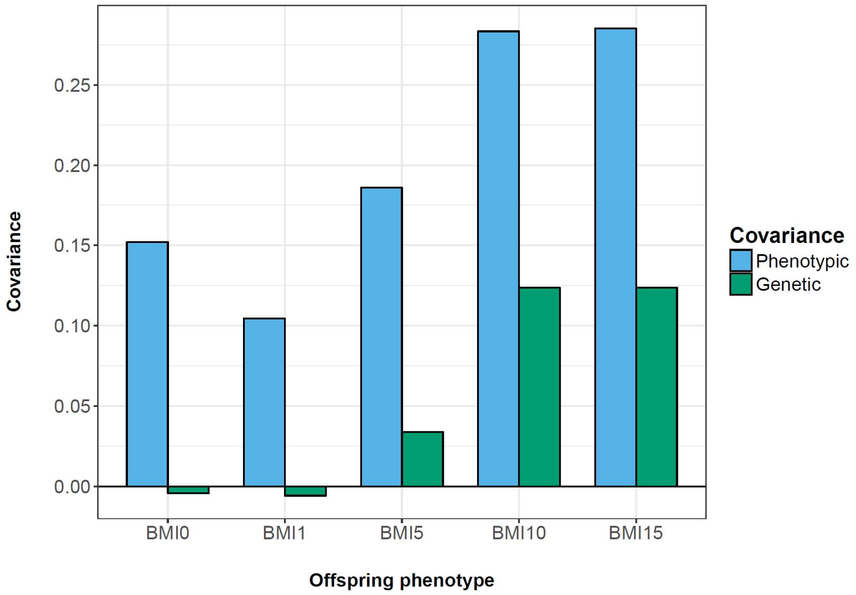

Figure S33: Partitioned covariance between maternal BMI and offspring ponderal index at all ages in the combined cohorts (pooled IPD estimates). **PI**: ponderal index at the indicated age (years)

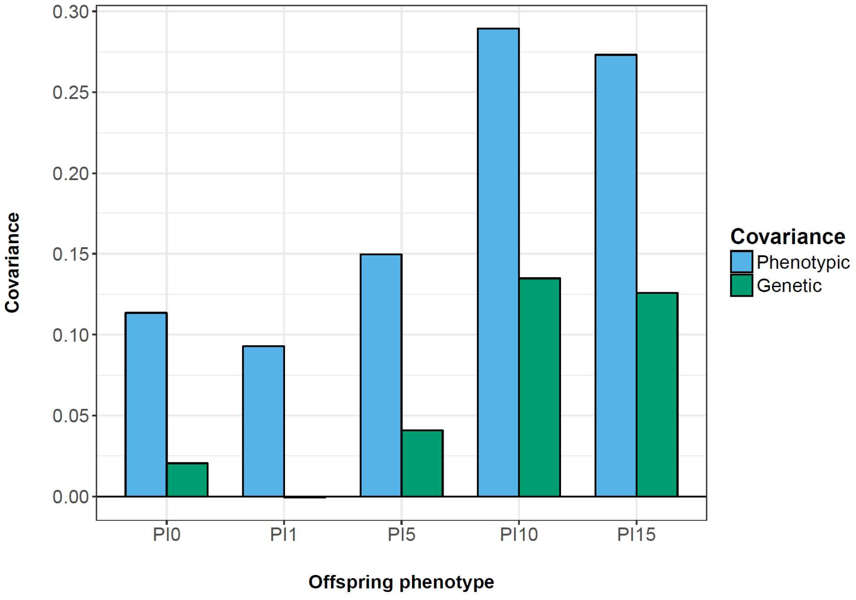

Note S34: Testing for inflation of heritability estimates due to population structure

We partitioned h^2_{SNP} by chromosome (29), as proposed by Yang *et al.* (30), to test for inflation of h^2_{SNP} estimates due to population structure (cryptic relatedness and population stratification). In brief, this involves splitting the genome into disjoint portions (in our case approximate halves [chromosomes 1-7 and 8-22]) and estimating h^2_{SNP} from each of these disjoint halves separately ($h^2_{\mathrm{CHR\ 1\text{-}7}}$ and $h^2_{\mathrm{CHR\ 8\text{-}22}}$). Inflation of h^2_{SNP} estimates due to population structure is estimated by the difference between the sum of h^2_{SNP} estimates from the disjoint halves and h^2_{SNP} estimated from the whole genome by fitting both halves jointly (h^2_{JOINT}). **Table S35** shows that estimated h^2_{SNP} due to population structure was negligible for the combined cohorts (pooled IPD estimates), providing reassurance that our results are unlikely to be substantively biased by cryptic relatedness or population stratification.

Table S35: Estimated SNP heritability explained by population structure for all phenotypes. $\boldsymbol{h^2_{\mathrm{JOINT}}}$: h^2_{SNP} estimates fitting both chromosome partitions (chromosomes 1-7 and 8-22) simultaneously, **SE**: standard errors of h^2_{SNP} estimates fitting chromosome partitions simultaneously, $\boldsymbol{h^2_{\mathrm{CHR\ 1\text{-}7}}}$: h^2_{SNP} estimates for chromosomes 1-7 only, $\boldsymbol{h^2_{\mathrm{CHR\ 8\text{-}22}}}$: h^2_{SNP} estimates for chromosomes 8-22 only, $\boldsymbol{h^2_{\mathrm{POP.\ STRUCTURE}}}$: estimated inflation of h^2_{SNP} estimates due to population structure (i.e. true h^2_{SNP} = h^2_{JOINT} - $h^2_{\mathrm{POP.\ STRUCTURE}}$).

Phenotype	h^2_{JOINT}	SE	$h^2_{\mathrm{CHR\ 1\text{-}7}}$	$h^2_{\mathrm{CHR\ 8\text{-}22}}$	$h^2_{\mathrm{POP.\ STRUCTURE}}$
Birth weight	0.274	0.031	0.114	0.169	0.010
1 year BMI	0.339	0.034	0.180	0.166	0.007
5 year BMI	0.260	0.036	0.116	0.142	-0.002
10 year BMI	0.353	0.037	0.142	0.210	-0.001
15 year BMI	0.277	0.036	0.102	0.171	-0.004

Note S36: Leave-one-out jackknife procedure

We used a leave-one-out jackknife procedure (31, 32) to calculate standard errors (SEs) and 95% confidence intervals (CIs) for Cov_G, Cov_P and Cov_G:Cov_P because to our knowledge, no formula is available for the SE of Cov_G:Cov_P, and non-parametric bootstrapping is not possible in this setting[a]. We first calculated N estimates of the parameter of interest (Cov_G, Cov_P or Cov_G:Cov_P) for each of the samples of size $N - 1$ obtained by omitting the Nth observation; these are referred to as partial estimates, denoted T_{-n}. We then calculated pseudovalues, denoted T_n^*, from each of the partial estimates as $T_n^* = NT - (N-1)T_{-n}$. We calculated the jackknife SE, denoted SE_{JACK}, as the standard deviation of the pseudovalues:

$$SE_{JACK} = \sqrt{\frac{\sum(T_n^* - \bar{T}_\bullet^*)^2}{N(N-1)}}, \qquad \text{(Equation 22)}$$

[a] Non-parametric bootstrapping involves sampling with replacement, which is not possible for GCTA-GREML analyses since individuals present more than once in a bootstrap sample would be genetically equivalent to monozygotic twins, therefore would be removed on application of a relatedness exclusion threshold to the GRM.

where $\bar{T}_{\bullet}^{*}$ is the mean of the pseudovalues. Finally, we calculated 95% CIs as the point estimate from the entire sample ± 1.96 * SE_{JACK}.

Note S37: Validation of jackknife procedure via simulation

We conducted a simulation to establish that confidence intervals (CIs) for a ratio of covariances calculated using a leave-one-out jackknife approach are likely to have good coverage properties in our case. The simulation involved four steps:

1. Simulation of a population of ten million mother-offspring dyads. Each dyad had two normally distributed genetic values and two normally distributed environmental values (i.e. a maternal genetic value, a maternal environmental value, an offspring genetic value and an offspring environmental value). We simulated this random vector of genetic and environmental values $\mathbf{x} = [g_1, e_1, g_2, e_2]^{\mathrm{T}}$ from a multivariate normal distribution: $\mathbf{x} \sim N(\boldsymbol{\mu}, \mathbf{V})$,

   where $\boldsymbol{\mu} = [0 \quad 0 \quad 0 \quad 0]^{\mathrm{T}}$, (Equation 23)

   $$\mathbf{V} = \begin{bmatrix} Var(g1) & 0 & Cov(g1, g2) & 0 \\ 0 & Var(e1) & 0 & Cov(e1, e2) \\ Cov(g1, g2) & 0 & Var(g2) & 0 \\ 0 & Cov(e1, e2) & 0 & Var(e2) \end{bmatrix}, \quad \text{(Equation 24)}$$

   g_1, e_1 and g_2, e_2 denote genetic and environmental values for the mother and offspring respectively and variance and covariance values were chosen to give values of Cov_P, Cov_G:Cov_P and h^2_{SNP} similar to our empirical results (**Table S38**). Two phenotypic values (one maternal value, one offspring value) were calculated for each dyad as the sum of the relevant maternal or offspring genetic and environmental values. Four sets of simulated parameters were used (**Table S38**)
2. Selection at random of m = 5000 samples, of size n = 250[b], from the simulated population
3. Calculation of Cov_G, Cov_P and Cov_G:Cov_P for each sample (using **Equation 1** in **Note S9**), along with 95% CIs for each parameter using a leave-one-out jackknife procedure (**Note S36**)
4. Calculation of CI coverage as the proportion of the m = 5000 confidence intervals that included the true population value

Simulation results (**Table S38**) suggested that confidence intervals from the leave-one-out jackknife have good coverage properties, including for a ratio of covariances such as Cov_G:Cov_P. In the simulation we also compared CI coverage to that from a non-parametric bootstrap approach (bias corrected and accelerated [BCa] bootstrap), and found that CI coverage was extremely similar for the jackknife and bootstrap (data available on request). We were able to use the bootstrap approach for our analyses of simulated data, but not for our

[b] Sample size was chosen in order to give standard errors for Cov_G of a similar magnitude to those from our primary GCTA-GREML analyses.

primary empirical analyses, because in the simulation we used **Equation 1** (**Note S9**) to calculate the genetic covariance, whereas in the empirical analyses we used GCTA-GREML.

Table S38: Simulation results and simulated parameters.

Simulation	Simulated parameters			95% CI coverage		
	Cov_P	Cov_G	Cov_G:Cov_P	Cov_G	Cov_P	Cov_G:Cov_P
1	0.3	0.15	0.5	0.943	0.948	0.940
2	0.3	0	0	0.947	0.949	0.968
3	0.15	0.075	0.5	0.947	0.951	0.911
4	0.15	0	0	0.947	0.952	0.986

Table S39: Associations between maternal BMI, potential confounders and offspring phenotypes

Associations of maternal pre-pregnancy BMI with potential confounders and offspring phenotypes, in the samples used for the primary analyses. † Continuous variables: regression coefficients from linear regression of variable on maternal BMI (both variables standardised, severely skewed variables natural logged), categorical variables: mean maternal BMI by category (95% CIs)

Variable	Cohort		Mean or N	SD or %	Association with maternal BMI†
Maternal BMI (kg/m2)	NFBC1966		23.0	3.3	
	NFBC1986		22.2	3.3	
	ALSPAC		22.9	3.8	
Maternal age (years)	NFBC1966		27.6	6.3	0.40 (0.37, 0.44)
	NFBC1986		28.0	5.3	0.22 (0.18, 0.27)
	ALSPAC		29.4	4.6	-0.01 (-0.03, 0.02)
Parity	NFBC1966	0	1028	35.6%	21.8 (21.7, 22.0)
		1	730	25.3%	22.7 (22.5, 22.9)
		2-3	733	25.4%	23.7 (23.5, 23.9)
		>=3	400	13.8%	25.6 (25.2, 26.0)
	NFBC1986	0	1428	68.2%	21.7 (21.6, 21.9)
		1	410	19.6%	23.0 (22.7, 23.4)
		2-3	166	7.9%	23.5 (22.9, 24.1)
		>=3	90	4.3%	24.3 (23.5, 25.1)
	ALSPAC	0	5184	81.7%	22.8 (22.7, 22.9)
		1	852	13.4%	23.2 (23.0, 23.5)
		2-3	290	4.6%	23.8 (23.3, 24.3)
		>=3	17	0.3%	23.4 (21.1, 25.8)
Socio-economic position	NFBC1966	I	280	10.0%	22.9 (22.5, 23.2)
		II	556	19.8%	23.0 (22.7, 23.3)
		III	1044	37.2%	22.7 (22.5, 22.9)
		IV	475	16.9%	23.1 (22.8, 23.4)
		Farmer I	245	8.7%	23.9 (23.4, 24.3)
		Farmer II	208	7.4%	24.5 (24.0, 25.1)
	NFBC1986	Professional/entrepreneur	340	18.8%	22.0 (21.7, 22.3)
		Skilled, non-manual	318	17.6%	22.3 (21.9, 22.6)
		Skilled, manual	743	41.0%	22.3 (22.1, 22.5)
		Unskilled/apprentice	87	4.8%	22.1 (21.2, 22.9)
		Farmer	143	7.9%	23.6 (22.9, 24.2)
		Student/at home	72	4.0%	21.2 (20.7, 21.7)
		Sick pension/unemployed	107	5.9%	22.8 (22.1, 23.5)
	ALSPAC	I	947	15.7%	22.1 (21.9, 22.3)
		II	2697	44.6%	22.8 (22.7, 22.9)
		III: manual	1470	24.3%	23.2 (23.0, 23.4)
		III: non-manual	651	10.8%	23.4 (23.1, 23.7)
		IV	249	4.1%	23.8 (23.2, 24.4)
		V	27	0.4%	24.5 (22.5, 26.4)

Table S39 (continued): Associations of maternal pre-pregnancy BMI with potential confounders and offspring phenotypes, in the samples used for the primary analyses. † Continuous variables: regression coefficients from linear regression of variable on maternal BMI (both variables standardised, severely skewed variables natural logged), categorical variables: mean maternal BMI by category (95% CIs)

Variable	Cohort		Mean or N	SD or %	Association with maternal BMI†
Maternal smoking	NFBC1966	No	2454	86.0%	23.1 (23.0, 23.3)
		Light	346	12.1%	22.4 (22.1, 22.8)
		Heavy	52	1.8%	22.9 (22.1, 23.8)
	NFBC1986	No	1666	81.5%	22.3 (22.1, 22.4)
		Light	180	8.8%	22.2 (21.7, 22.7)
		Heavy	198	9.7%	22.2 (21.7, 22.6)
	ALSPAC	Never during pregnancy	4435	72.1%	22.9 (22.8, 23.0)
		Early pregnancy only	624	10.1%	23.1 (22.8, 23.4)
		Throughout pregnancy	1096	17.8%	22.8 (22.6, 23.0)
Gestational age (weeks)	NFBC1966	<37	123	4.3%	22.7 (22.2, 23.3)
		37-40	737	25.5%	22.9 (22.7, 23.1)
		40-42	1443	49.9%	23.1 (23.0, 23.3)
		>=42	591	20.4%	23.1 (22.8, 23.4)
	NFBC1986	<37	31	1.5%	22.4 (21.2, 23.6)
		37-40	608	29.0%	22.1 (21.8, 22.3)
		40-42	1245	59.5%	22.3 (22.1, 22.5)
		>=42	210	10.0%	22.4 (22.0, 22.9)
	ALSPAC	<37	294	4.5%	22.9 (22.4, 23.4)
		37-40	2450	37.6%	22.9 (22.8, 23.1)
		40-42	3289	50.5%	22.9 (22.8, 23.0)
		>=42	477	7.3%	23.1 (22.7, 23.4)
Birth weight (g)	NFBC1966		3510	520	0.22 (0.18, 0.25)
	NFBC1986		3610	490	0.19 (0.15, 0.23)
	ALSPAC		3450	520	0.13 (0.11, 0.15)
Birth length (cm)	NFBC1966		50.4	2.1	0.15 (0.11, 0.18)
	NFBC1986		50.7	2.1	0.10 (0.06, 0.15)
	ALSPAC		50.8	2.3	0.06 (0.04, 0.09)
1yr BMI (kg/m2)	NFBC1966		17.8	1.6	0.13 (0.09, 0.17)
	NFBC1986		17.3	1.4	0.09 (0.05, 0.14)
	ALSPAC		17.5	1.5	0.09 (0.06, 0.11)
5yr BMI (kg/m2)	NFBC1966		15.5	1.4	0.16 (0.12, 0.20)
	NFBC1986		15.8	1.5	0.19 (0.15, 0.24)
	ALSPAC		16.2	1.5	0.19 (0.17, 0.22)
10yr BMI (kg/m2)	NFBC1966		17.0	2.3	0.22 (0.18, 0.26)
	NFBC1986		17.6	2.8	0.25 (0.20, 0.29)
	ALSPAC		17.7	2.8	0.32 (0.30, 0.35)
15yr BMI (kg/m2)	NFBC1966		19.7	2.7	0.22 (0.19, 0.26)
	NFBC1986		21.3	3.7	0.27 (0.22, 0.31)
	ALSPAC		21.0	3.5	0.35 (0.33, 0.38)

Table S40: Phenotypic and genetic covariance at all ages in the combined cohorts

Estimates of phenotypic covariance (Cov_P), genetic covariance (Cov_G) and the ratio of Cov_G to Cov_P, between maternal BMI and offspring phenotype, from the combined cohorts (pooled IPD estimates) and from NFBC1966 for adult BMI. All variables were standardised to give mean zero and variance one, therefore phenotypic covariances are equivalent to Pearson correlation coefficients.

Offspring phenotype	N	Cov_P	(95% CI)	Cov_G	(95% CI)	Cov_G:Cov_P	(95% CI)
Birth weight	11498	0.15	(0.13, 0.17)	-0.04	(-0.09, 0.01)	-0.26	(-0.63, 0.10)
1yr BMI	10733	0.10	(0.08, 0.12)	0.00	(-0.07, 0.06)	-0.05	(-0.68, 0.59)
5yr BMI	9915	0.19	(0.16, 0.21)	0.03	(-0.04, 0.10)	0.18	(-0.21, 0.56)
10yr BMI	9433	0.28	(0.25, 0.31)	0.12	(0.04, 0.20)	0.44	(0.16, 0.71)
15yr BMI	9875	0.29	(0.26, 0.31)	0.12	(0.04, 0.21)	0.43	(0.15, 0.72)
31yr BMI	3711	0.18	(0.15, 0.22)	0.23	(0.06, 0.39)	1.25	(0.35, 1.37)
46yr BMI	3079	0.16	(0.13, 0.20)	0.13	(-0.08, 0.33)	0.78	(-0.46, 1.87)

Note S41: Standard meta-analysis results

For the primary analyses we merged individual participant data (IPD) from the three cohorts and fitted the GCTA-GREML model on the pooled dataset. In the meta-analysis literature this is referred to as one-stage IPD meta-analysis (33), and has also been referred to as mega-analysis, however for simplicity we use the term “pooled IPD estimates” here. We standardised phenotypic variables in the combined cohorts after merging. These pooled IPD estimates had greater statistical efficiency than a standard meta-analysis in which the GCTA-GREML model is fitted separately for each cohort, and the pooled effect is then estimated using a fixed or random effects model, but assumed that the three cohorts were from the same population. As a sensitivity analysis we therefore conducted a standard meta-analysis: we calculated estimates within each cohort separately (having previously carried out standardisation of phenotypic variables within separate cohorts), followed by estimation of the pooled effect using both fixed (inverse variance weighted) and random effects (DerSimonian and Laird (34)) models, implemented with the *metan* command (35) in Stata. These standard meta-analyses tested whether results were sensitive to **(1)** standardisation of phenotypes within cohorts separately, and **(2)** for the random effects model, relaxation of the assumption that the three cohorts are from the same population.

Table S42 shows heterogeneity statistics from the standard meta-analysis. There was marked heterogeneity for Cov_P at most ages, which may have been due to differences in the strength of the obesogenic environment between cohorts, but less evidence of heterogeneity for Cov_G and Cov_G:Cov_P, particularly at birth, 10 years and 15 years. **Tables S43** and **S44,** and **Figures S45** and **S46** show that results from the standard meta-analyses using both the fixed and random effects models were similar to our primary pooled IPD estimates, albeit with wider confidence intervals. The similarity of pooled IPD estimates to those from the random effects standard meta-analysis suggests that the former are robust to relaxation of the assumption that the three cohorts are from the same population, and to standardisation of phenotypic variables separately within cohorts. **Table S47** gives results from the separate cohorts, which were input into the standard meta-analysis.

Table S42: Meta-analysis heterogeneity statistics for phenotypic covariance (Cov_P), genetic covariance (Cov_G) and Cov_G:Cov_P between maternal BMI and offspring phenotype for the three cohorts. *P*-values are from a chi-squared test for heterogeneity

Offspring phenotype	Cov_P		Cov_G		Cov_G:Cov_P	
	I^2	*P*-value	I^2	*P*-value	I^2	*P*-value
Birth weight	85.4%	0.001	0.0%	0.523	0.0%	0.422
1 year BMI	42.6%	0.175	57.8%	0.093	54.6%	0.111
5 year BMI	11.8%	0.322	21.5%	0.280	24.0%	0.268
10 year BMI	90.2%	<0.001	0.0%	0.920	0.0%	0.753
15 year BMI	93.2%	<0.001	0.0%	0.602	0.0%	0.380

Table S43: Standard meta-analysis results for phenotypic covariance (Cov_P), genetic covariance (Cov_G) and Cov_G:Cov_P between maternal BMI and offspring phenotype for the three cohorts, from the fixed effects model (inverse variance weighted)

Offspring phenotype	N	Cov_P (95% CI)	Cov_G (95% CI)	Cov_G:Cov_P (95% CI)
Birth Weight	13001	0.17 (0.15, 0.18)	-0.04 (-0.12, 0.04)	-0.14 (-0.61, 0.33)
1yr BMI	12074	0.10 (0.08, 0.12)	0.01 (-0.08, 0.10)	0.28 (-0.59, 1.14)
5yr BMI	10948	0.19 (0.16, 0.21)	0.02 (-0.08, 0.12)	0.09 (-0.43, 0.61)
10yr BMI	10470	0.26 (0.24, 0.29)	0.11 (0.00, 0.22)	0.35 (-0.02, 0.72)
15yr BMI	11341	0.28 (0.25, 0.30)	0.13 (0.02, 0.23)	0.39 (0.05, 0.72)

Table S44: Standard meta-analysis results for phenotypic covariance (Cov_P), genetic covariance (Cov_G) and Cov_G:Cov_P between maternal BMI and offspring phenotype for the three cohorts, from the random effects model (DerSimonian and Laird)

Offspring phenotype	N	Cov_P (95% CI)	Cov_G (95% CI)	Cov_G:Cov_P (95% CI)
Birth Weight	13001	0.17 (0.12, 0.22)	-0.04 (-0.12, 0.04)	-0.14 (-0.61, 0.33)
1yr BMI	12074	0.10 (0.08, 0.13)	0.03 (-0.11, 0.17)	0.25 (-1.07, 1.57)
5yr BMI	10948	0.19 (0.16, 0.21)	0.04 (-0.08, 0.16)	0.19 (-0.48, 0.87)
10yr BMI	10470	0.26 (0.18, 0.35)	0.11 (0.00, 0.22)	0.35 (-0.02, 0.72)
15yr BMI	11341	0.28 (0.19, 0.38)	0.13 (0.02, 0.23)	0.39 (0.05, 0.72)

Figure S45: Standard meta-analysis results for phenotypic covariance (Cov_P), genetic covariance (Cov_G) and Cov_G:Cov_P between maternal BMI and offspring phenotype for the three cohorts, from the fixed effects model (inverse variance weighted)

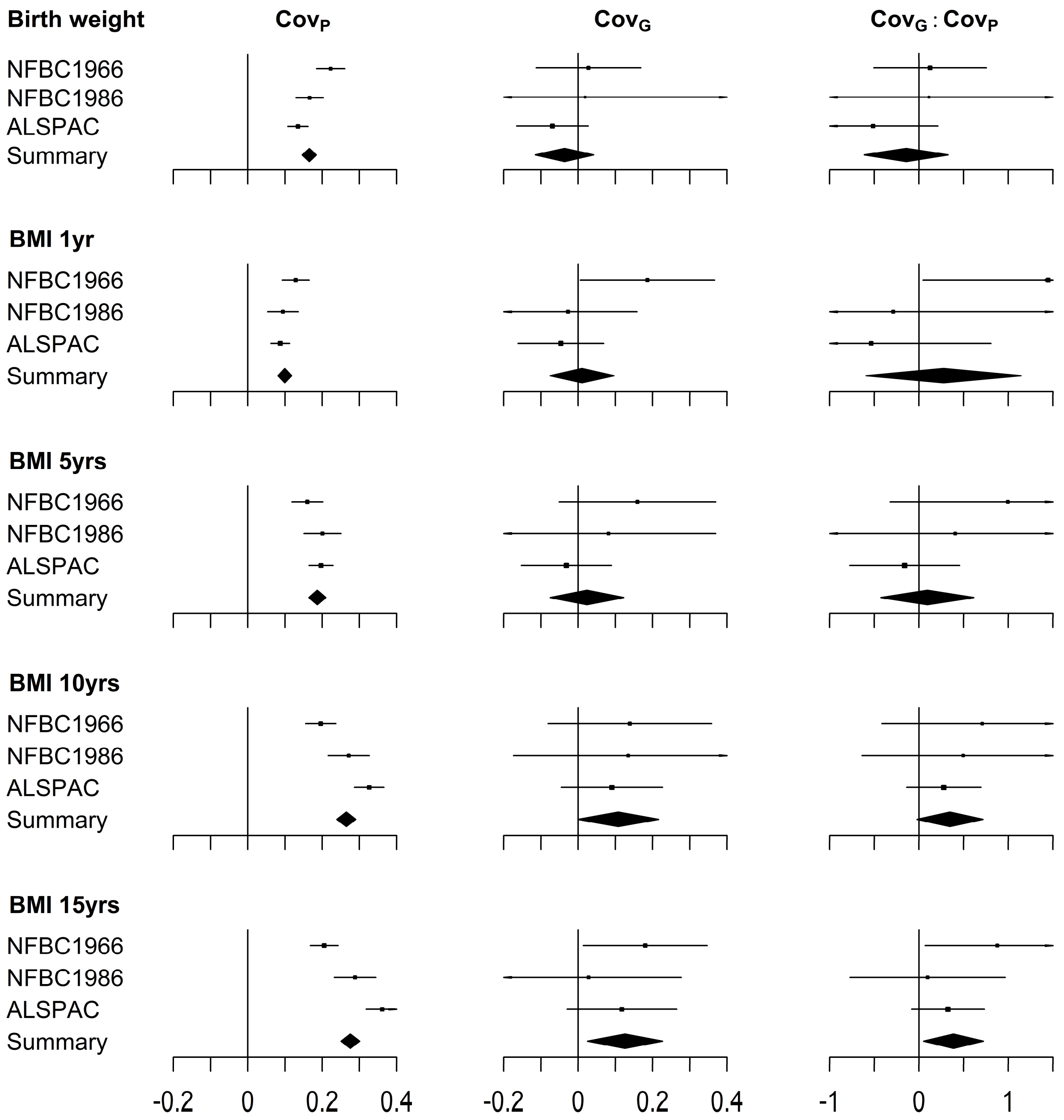

Figure S46: Standard meta-analysis results for phenotypic covariance (Cov_P), genetic covariance (Cov_G) and Cov_G:Cov_P between maternal BMI and offspring phenotype for the three cohorts, from the random effects model (DerSimonian and Laird)

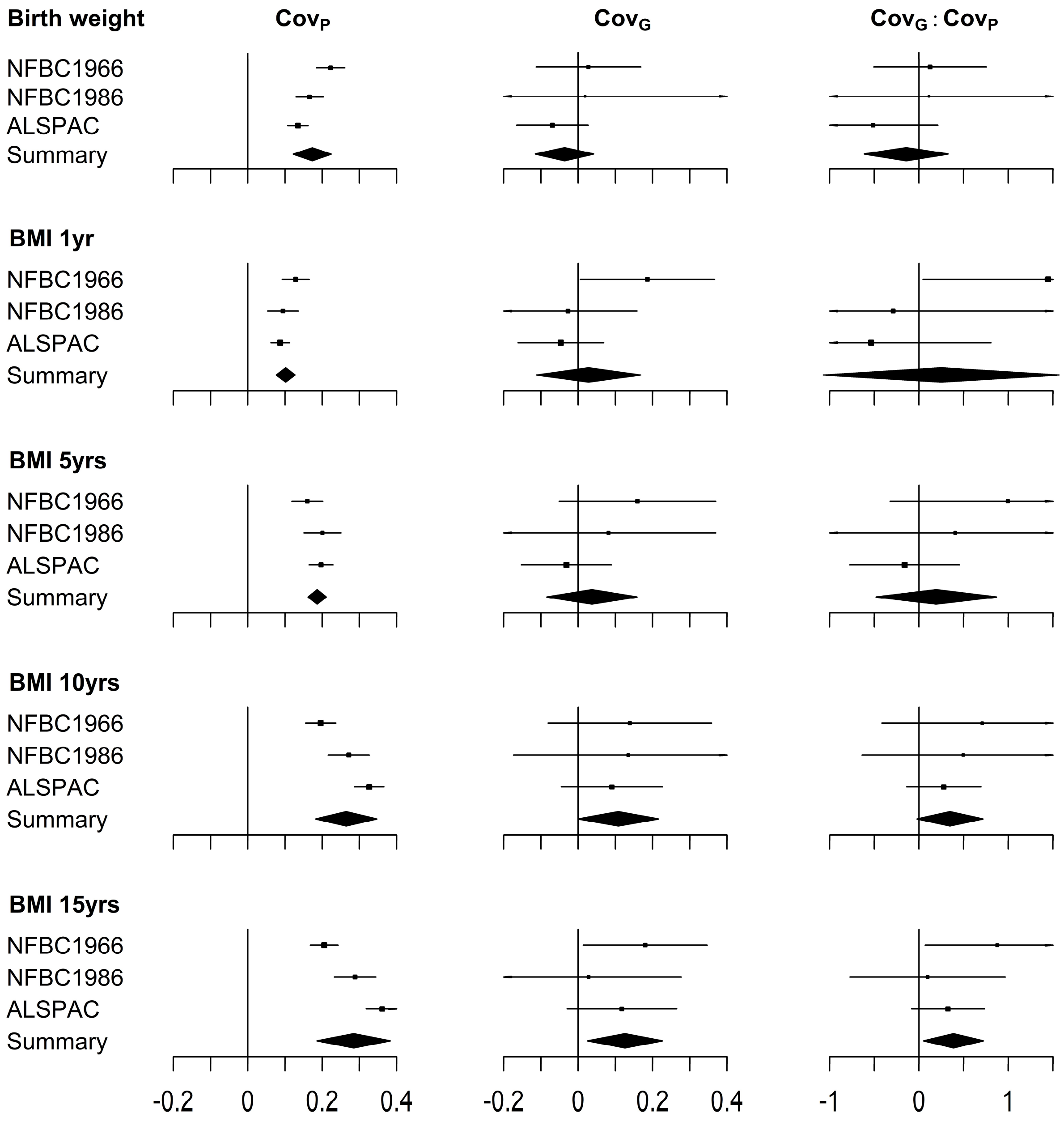

Table S47: Estimates of phenotypic covariance (Cov_P), genetic covariance (Cov_G) and Cov_G:Cov_P between maternal BMI and offspring phenotype for the separate cohorts

Cohort	Offspring phenotype	N	Cov_P			Cov_G			Cov_G:Cov_P		
			Estimate	95% CI		Estimate	95% CI		Estimate	95% CI	
NFBC1966	Birth weight	3702	0.22	0.18	0.26	0.03	-0.11	0.17	0.12	-0.51	0.75
	1 year BMI	3450	0.13	0.09	0.16	0.19	0.01	0.37	1.45	0.05	2.85
	5 year BMI	2692	0.16	0.12	0.20	0.16	-0.05	0.37	0.99	-0.32	2.31
	10 year BMI	2706	0.20	0.16	0.24	0.14	-0.08	0.36	0.71	-0.42	1.83
	15 year BMI	3665	0.21	0.17	0.24	0.18	0.01	0.35	0.88	0.07	1.68
	31 year BMI	3711	0.18	0.15	0.22	0.23	0.06	0.39	1.25	0.35	2.16
	46 year BMI	3079	0.16	0.13	0.20	0.13	-0.08	0.33	0.78	-0.46	2.02
NFBC1986	Birth weight	2719	0.17	0.13	0.20	0.02	-0.46	0.50	0.11	-2.76	2.99
	1 year BMI	2400	0.09	0.05	0.14	-0.03	-0.21	0.16	-0.29	-2.25	1.68
	5 year BMI	2265	0.20	0.15	0.25	0.08	-0.21	0.37	0.41	-1.03	1.84
	10 year BMI	2221	0.27	0.22	0.33	0.13	-0.17	0.44	0.50	-0.64	1.63
	16 year BMI	2732	0.29	0.23	0.34	0.03	-0.22	0.28	0.10	-0.77	0.97
ALSPAC	Birth weight	6580	0.13	0.11	0.16	-0.07	-0.17	0.03	-0.51	-1.24	0.21
	1 year BMI	6224	0.09	0.06	0.11	-0.05	-0.16	0.07	-0.54	-1.88	0.81
	4 year BMI	5991	0.20	0.16	0.23	-0.03	-0.15	0.09	-0.16	-0.78	0.45
	10 year BMI	5543	0.33	0.29	0.37	0.09	-0.05	0.23	0.28	-0.14	0.69
	15 year BMI	4944	0.36	0.32	0.40	0.12	-0.03	0.26	0.32	-0.08	0.73

Note S48: Examining the effect of missing data

Missing data caused the samples used for our primary GCTA-GREML analyses to be smaller than the full sample of live born singletons at baseline (**Note S3**, **Figure S4**). A substantial proportion of individuals (particularly in the NFBCs) had missing genotype data (**Figure S4**), and some individuals with genotype available had to be excluded due to cryptic relatedness (**Table S50**). Furthermore, although phenotype data were available for most individuals at birth, the proportion with missing phenotype data increased with age. It is possible that missing data could result in selection bias, for both the phenotypic and genetic covariance. To explore the potential for selection bias we examined the phenotypic associations between maternal BMI and offspring BW in the samples of live born singletons at baseline, and compared this to the same association in the samples used for the GCTA-GREML analyses (pooled IPD estimates). **Supplementary Table S49** shows that there were not substantial differences in the phenotypic associations between the baseline samples and the GCTA-GREML samples, suggesting that selection bias is unlikely to be large, at least for the phenotypic associations. We were unable to perform similar comparisons for the genetic covariance or for the phenotypic covariance at later ages, because due to the missing data it would be impossible to estimate these parameters in the baseline sample.

Table S49: Results from linear regression of offspring BW (*z*-score) on maternal BMI (*z*-score), for the full sample of live born singletons at baseline with non-missing data for maternal BMI and offspring BW, and for the samples used for the primary GCTA-GREML analyses. Both variables were standardised, therefore the regression coefficient is equivalent to both Pearson's correlation coefficient and to the covariance

**Cohort**		**Full sample at baseline**	**Sample for primary GCTA-GREML analyses**				
			**Birth**	**1 year**	**5 years**	**10 years**	**15 years**
NFBC1966	N	10602	2894	2736	2145	2146	2866
	Beta (95% CI)	0.21 (0.19, 0.23)	0.22 (0.18, 0.25)	0.21 (0.18, 0.25)	0.21 (0.17, 0.25)	0.22 (0.18, 0.27)	0.23 (0.20, 0.27)
NFBC1986	N	8972	2094	1838	1840	1793	2107
	Beta (95% CI)	0.16 (0.14, 0.18)	0.19 (0.15, 0.23)	0.17 (0.13, 0.22)	0.18 (0.13, 0.22)	0.17 (0.12, 0.21)	0.19 (0.15, 0.23)
ALSPAC	N	11120	6510	6159	5930	5494	4902
	Beta (95% CI)	0.14 (0.13, 0.16)	0.13 (0.11, 0.15)	0.13 (0.10, 0.15)	0.13 (0.10, 0.15)	0.14 (0.11, 0.16)	0.14 (0.11, 0.17)

Table S50: Number of individuals removed at varying relatedness exclusion thresholds

Number of individuals removed at different relatedness cut-offs, for the samples of live born singletons with genotype data available (prior to exclusion of individuals with missing phenotype data). The numbers for the combined cohorts are less than the sum of those for the separate cohorts, because in a larger sample there is more potential for any given individual to be closely related to another individual in the sample

Cohort	Relatedness threshold	N remaining	N removed	% removed
**Combined cohorts**	0.025	7483	9764	56.6%
	0.05	12917	4330	25.1%
	0.075	14497	2750	15.9%
	0.1	15198	2049	11.9%
	0.125	15729	1518	8.8%
	0.15	16352	895	5.2%
	0.175	16804	443	2.6%
	0.2	16912	335	1.9%
**NFBC1966**	0.025	2335	2972	56.0%
	0.05	3997	1310	24.7%
	0.075	4502	805	15.2%
	0.1	4690	617	11.6%
	0.125	4905	402	7.6%
	0.15	5160	147	2.8%
	0.175	5290	17	0.3%
	0.2	5305	2	<0.1%
**NFBC1986**	0.025	1953	1392	41.6%
	0.05	2783	562	16.8%
	0.075	2971	374	11.2%
	0.1	3049	296	8.8%
	0.125	3153	192	5.7%
	0.15	3264	81	2.4%
	0.175	3329	16	0.5%
	0.2	3343	2	0.1%
**ALSPAC**	0.025	6709	1886	21.9%
	0.05	7758	837	9.7%
	0.075	7956	639	7.4%
	0.1	8057	538	6.3%
	0.125	8259	336	3.9%
	0.15	8462	133	1.5%
	0.175	8549	46	0.5%
	0.2	8566	29	0.3%

Abbreviations

Abbreviation	Meaning
ALSPAC	Avon Longitudinal Study of Parents and Children
BCa bootstrap	Bias corrected and accelerated bootstrap
BMI	Body mass index
BW	Birth weight
CI	Confidence interval
CNV	Copy number variation
Cov_G	Genetic covariance (i.e. the part of the phenotypic covariance explained by additive genetic effects as captured by imputed SNPs)
Cov_P	Phenotypic covariance
GCTA	Genome-wide Complex Trait Analysis
GCTA-GREML	Genomic-relatedness-based Restricted Maximum Likelihood implemented in the GCTA software
GRM	Genetic relatedness matrix
h^2_{SNP}	SNP heritability
HWE	Hardy-Weinberg equilibrium
IBD	Identity by descent
IPD	Individual participant data
MAC	Minor allele count
MAF	Minor allele frequency
MDS	Multidimensional scaling
NFBC	Northern Finland Birth Cohort
PC	Principal component
PI	Ponderal index
r_G	Genetic correlation
SE	Standard error
SEP	Socioeconomic position
SNP	Single nucleotide polymorphism
Var_G	Genetic variance (i.e. the part of the phenotypic variance explained by additive genetic effects as captured by imputed SNPs)
Var_P	Phenotypic variance
WHO	World Health Organisation

References

1. Koupil I, Toivanen P. Social and early-life determinants of overweight and obesity in 18-year-old Swedish men. *Int J Obes*. 2008;32(1):73.
2. Fujita Y, Kouda K, Nakamura H, Iki M. Relationship Between Maternal Pre-pregnancy Weight and Offspring Weight Strengthens as Children Develop: A Population-Based Retrospective Cohort Study. *J Epidemiol*. 2018:JE20170137.
3. West J, Santorelli G, Whincup PH, *et al.* Association of maternal exposures with adiposity at age 4/5 years in white British and Pakistani children: findings from the Born in Bradford study. *Diabetologia*. 2018;61(1):242-52.

4. Veena SR, Krishnaveni GV, Karat SC, Osmond C, Fall CH. Testing the fetal overnutrition hypothesis; the relationship of maternal and paternal adiposity to adiposity, insulin resistance and cardiovascular risk factors in Indian children. *Public Health Nutr*. 2013;16(9):1656-66.
5. Widen EM, Whyatt RM, Hoepner LA*, et al.* Gestational weight gain and obesity, adiposity and body size in African–American and Dominican children in the Bronx and Northern Manhattan. *Matern Child Nutr*. 2016;12(4):918-28.
6. Zalbahar N, Najman J, McIntrye HD, Mamun A. Parental pre-pregnancy BMI influences on offspring BMI and waist circumference at 21 years. *Aust N Z J Public Health*. 2016;40(6):572-8.
7. Ouyang F, Parker MG, Luo ZC*, et al.* Maternal BMI, gestational diabetes, and weight gain in relation to childhood obesity: the mediation effect of placental weight. *Obesity*. 2016;24(4):938-46.
8. Castillo H, Santos IS, Matijasevich A. Relationship between maternal pre-pregnancy body mass index, gestational weight gain and childhood fatness at 6–7 years by air displacement plethysmography. *Matern Child Nutr*. 2015;11(4):606-17.
9. Gademan MG, Vermeulen M, Oostvogels AJ*, et al.* Maternal prepregancy BMI and lipid profile during early pregnancy are independently associated with offspring's body composition at age 5–6 years: the ABCD study. *PLoS One*. 2014;9(4):e94594.
10. Gaillard R, Steegers EA, Duijts L*, et al.* Childhood cardiometabolic outcomes of maternal obesity during pregnancy the generation R study. *Hypertension*. 2014;63(4):683-91.
11. Fleten C, Nystad W, Stigum H*, et al.* Parent-offspring body mass index associations in the Norwegian Mother and Child Cohort Study: a family-based approach to studying the role of the intrauterine environment in childhood adiposity. *Am J Epidemiol*. 2012;176(2):83-92.
12. Lawlor DA, Lichtenstein P, Långström N. Association of Maternal Diabetes Mellitus in Pregnancy With Offspring Adiposity Into Early Adulthood: Clinical Perspective. *Circulation*. 2011;123(3):258-65.
13. Reynolds R, Osmond C, Phillips D, Godfrey K. Maternal BMI, parity, and pregnancy weight gain: influences on offspring adiposity in young adulthood. *J Clin Endocrinol Metab*. 2010;95(12):5365-9.
14. Lawlor DA, Timpson NJ, Harbord RM*, et al.* Exploring the developmental overnutrition hypothesis using parental–offspring associations and FTO as an instrumental variable. *PLoS Med*. 2008;5(3):e33.
15. Rantakallio P. Groups at risk in low birth weight infants and perinatal mortality. *Acta Paediatr Scand*. 1969;193:Suppl 193: 1+.
16. Järvelin MR, Hartikainen-Sorri AL, Rantakallio P. Labour induction policy in hospitals of different levels of specialisation. *BJOG*. 1993;100(4):310-5.
17. Northern Finland Birth Cohorts. Northern Finland Birth Cohorts [Available from: http://www.oulu.fi/nfbc/.
18. Boyd A, Golding J, Macleod J*, et al.* Cohort profile: the 'children of the 90s'—the index offspring of the Avon Longitudinal Study of Parents and Children. *Int J Epidemiol*. 2013;42(1):111-27.
19. Avon Longitudinal Study of Pregnancy and Childhood. Explore data and samples [Available from: http://www.bristol.ac.uk/alspac/researchers/our-data/.
20. Visscher PM, Yang J, Goddard ME. A commentary on 'common SNPs explain a large proportion of the heritability for human height'by Yang et al.(2010). *Twin Res Hum Genet*. 2010;13(6):517-24.
21. Sabatti C, Hartikainen A-L, Pouta A*, et al.* Genome-wide association analysis of metabolic traits in a birth cohort from a founder population. *Nat Genet*. 2009;41(1):35-46.
22. Plomin R, DeFries J. Multivariate behavioral genetic analysis of twin data on scholastic abilities. *Behav Genet*. 1979;9(6):505-17.

23. Yang J, Benyamin B, McEvoy BP*, et al.* Common SNPs explain a large proportion of the heritability for human height. *Nat Genet*. 2010;42(7):565-9.

24. Lee SH, Yang J, Goddard ME, Visscher PM, Wray NR. Estimation of pleiotropy between complex diseases using single-nucleotide polymorphism-derived genomic relationships and restricted maximum likelihood. *Bioinformatics*. 2012;28(19):2540-2.

25. Deary IJ, Yang J, Davies G*, et al.* Genetic contributions to stability and change in intelligence from childhood to old age. *Nature*. 2012;482(7384):212.

26. Yang J, Lee SH, Goddard ME, Visscher PM. GCTA: a tool for genome-wide complex trait analysis. *Am J Hum Genet*. 2011;88(1):76-82.

27. Evans LM, Tahmasbi R, Vrieze SI*, et al.* Comparison of methods that use whole genome data to estimate the heritability and genetic architecture of complex traits. *Nat Genet*. 2018;26:26.

28. Dandine-Roulland C, Bellenguez C, Debette S, Amouyel P, Génin E, Perdry H. Accuracy of heritability estimations in presence of hidden population stratification. *Sci Rep*. 2016;6.

29. Speed D, Cai N, The UCLEB Consortium, Johnson M, Nejentsev S, Balding D. Reevaluation of SNP heritability in complex human traits. *Nat Genet*. 2017.

30. Yang J, Manolio TA, Pasquale LR*, et al.* Genome partitioning of genetic variation for complex traits using common SNPs. *Nat Genet*. 2011;43(6):519-25.

31. Shao J, Tu D. *The jackknife and bootstrap*: Springer Science & Business Media; 2012.

32. Abdi H, Williams LJ. Jackknife. In: Neil S, editor. *Encyclopedia of Research Design*. Thousand Oaks, CA: Sage; 2010.

33. Riley RD, Lambert PC, Abo-Zaid G. Meta-analysis of individual participant data: rationale, conduct, and reporting. *BMJ*. 2010;340:c221.

34. DerSimonian R, Laird N. Meta-analysis in clinical trials. *Control Clin Trials*. 1986;7(3):177-88.

35. Harris R, Bradburn M, Deeks J, Harbord R, Altman D, Sterne J. Metan: fixed-and random-effects meta-analysis. *Stata J*. 2008;8(1):3.
